# Direct and Inverse Spin Splitting Effects in Altermagnetic RuO_2_

**DOI:** 10.1002/advs.202400967

**Published:** 2024-04-16

**Authors:** Yaqin Guo, Jing Zhang, Zengtai Zhu, Yuan‐yuan Jiang, Longxing Jiang, Chuangwen Wu, Jing Dong, Xing Xu, Wenqing He, Bin He, Zhiheng Huang, Luojun Du, Guangyu Zhang, Kehui Wu, Xiufeng Han, Ding‐fu Shao, Guoqiang Yu, Hao Wu

**Affiliations:** ^1^ Songshan Lake Materials Laboratory Dongguan Guangdong 523808 China; ^2^ Key Laboratory of Materials Physics Institute of Solid State Physics HFIPS Chinese Academy of Sciences Hefei 230031 China; ^3^ University of Science and Technology of China Hefei 230026 China; ^4^ Beijing National Laboratory for Condensed Matter Physics Institute of Physics Chinese Academy of Sciences Beijing 100190 China

**Keywords:** altermagnetic materials, inverse spin splitting effect, spin pumping, spin splitting effect, spin–torque ferromagnetic resonance

## Abstract

Recently, the altermagnetic materials with spin splitting effect (SSE), have drawn significant attention due to their potential to the flexible control of the spin polarization by the Néel vector. Here, the direct and inverse altermagnetic SSE (ASSE) in the (101)‐oriented RuO_2_ film with the tilted Néel vector are reported. First, the spin torque along the *x*‐, *y*‐, and *z*‐axis is detected from the spin torque‐induced ferromagnetic resonance (ST‐FMR), and the *z*‐spin torque emerges when the electric current is along the [010] direction, showing the anisotropic spin splitting of RuO_2_. Further, the current‐induced modulation of damping is used to quantify the damping‐like torque efficiency (*ξ*_DL_) in RuO_2_/Py, and an anisotropic *ξ*_DL_ is obtained and maximized for the current along the [010] direction, which increases with the reduction of the temperature, indicating the present of ASSE. Next, by way of spin pumping measurement, the inverse altermagnetic spin splitting effect (IASSE) is studied, which also shows a crystal direction‐dependent anisotropic behavior and temperature‐dependent behavior. This work gives a comprehensive study of the direct and inverse ASSE effects in the altermagnetic RuO_2_, inspiring future altermagnetic materials and devices with flexible control of spin polarization.

## Graphical abstract

The direct and inverse altermagnetic spin splitting effects (ASSE) in the (101)‐oriented RuO_2_ film with the tilted Néel vector, as well as the crystal direction‐dependent anisotropic behavior, confirm the presence of ASSE in the (101)‐RuO_2_. Furthermore, the observation of temperature‐dependent characteristics of ASSE and spin pumping signals confirm a dominated spin splitting in RuO_2_ at lower temperatures.

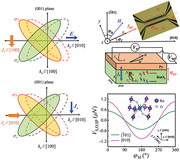

## 1 Introduction

Spintronic materials and devices have potential applications in the next‐generation low‐power and high‐speed memory and logic technology, such as magnetic random‐access memory (MRAM).^[^ ^1^, ^2^ ^]^ The crucial question of spintronics is how to efficiently generate the spin current by electrical methods. Spin transfer torque (STT), i.e., the electrical current can be polarized by the local magnetization in the magnetic layer (***σ*** // ***m***) and thus the spin‐polarized current can be used to exert a torque for another magnetic layer for magnetization switching or oscillation, which has been adopted in current STT‐MRAM technology.^[^ ^3^, ^4^ ^]^ Spin–orbit torque (SOT) is another phenomenon that typically generates the transversal spin current (***J*** *_s_*) by spin–orbit coupling with the spin‐polarized direction ***σ*** // ***J*** *_e_* × ***J*** *_s_*, which has been demonstrated in abundant material systems such as heavy metals,^[^ ^5^, ^6^, ^7^ ^]^ topological insulators,^[^ ^8^, ^9^, ^10^, ^11^, ^12^, ^13^ ^]^ oxide materials,^[^ ^14^ ^]^ and so on.^[^ ^15^, ^16^, ^17^, ^18^, ^19^ ^]^ SOT‐MRAM can separate the writing and reading paths and thus has an improved device endurance.^[^ ^20^, ^21^ ^]^

Generally, antiferromagnets are considered to possess negligible spin polarization due to the fully‐cancelled spin sub‐bands. Recently, the theoretical work predicts a new class of altermagnets by decoupling the spin and crystal spaces, with the zero net magnetization and an alternating spin‐momentum locking (spin splitting effect, SSE) in energy bands originating from the inequivalent crystal environments for two spin sublattices, which can provide a spin current with the polarization aligned along the Néel vector ***N*** and an induced spin splitting torque (SST) to the adjacent magnetization.^[^ ^22^, ^23^, ^24^, ^25^, ^26^, ^27^, ^28^ ^]^ Recently, researchers experimentally observed the spin splitting band structures of altermagnets, MnTe_2_^[^ ^29^ ^]^ and MnTe,^[^ ^30^ ^]^ by using the spin‐resolved and angle‐resolved photoemission spectroscopy (SARPES) and angle‐resolved photoemission spectroscopy (ARPES) measurements, respectively. These observations are consistent with the band structures theoretically predicted. Altermagnetism candidates are diverse, including quasi‐2D oxide insulator V_2_Se_2_O, 3D rutile fluoride or oxide insulators FeF_2_,^[^ ^31^ ^]^ MnO_2_,^[^ ^32^ ^]^ and metal RuO_2_.^[^ ^26^ ^]^ Additionally, materials like pnictide with metal–insulator transition FeSb_2_^[^ ^33^ ^]^ and metal CrSb^[^ ^22^ ^]^ among others,^[^ ^26^, ^34^, ^35^ ^]^ are also noteworthy candidates for altermagnetism. SSE in the altermagnets possesses the controllable spin polarization direction (*σ*) and controllable spin current flow direction (***J*** *_s_*) simultaneously by tuning the Néel vector ***N*** and the electric current ***J*** *_e_* directions, respectively, and therefore providing a more flexible way for the electrical control of magnetic order, such as an out‐of‐plane spin component for field‐free switching of the perpendicular magnet.

Ruthenium dioxide (RuO_2_) is a conductive rutile oxide with a collinear antiferromagnetic structure, exhibiting a high Néel temperature (*T*_N_ > 300 K).^[^ ^36^ ^]^ A recent study^[^ ^37^ ^]^ has introduced a dynamic approach to characterize the antiferromagnetic ordering in RuO_2_, unveiling a spatially periodic spin structure. Additionally, an anomalous Hall effect in RuO_2_ was observed,^[^ ^38^ ^]^ with an anomalous Hall conductivity exceeding 1000 Ω^−1^ cm^−1^ in thin films along different crystal orientations. Further, the research^[^ ^39^ ^]^ delves into the crystal thermal transport phenomena in RuO_2_, which result from the breaking of crystal time‐reversal symmetry. Based on the RuO_2_/Py heterostructures, several experimental works have observed the altermagnetic spin splitting effect (ASSE) in the altermagnetic RuO_2_‐based structures by the spin–torque ferromagnetic resonance (ST‐FMR)^[^ ^40^, ^41^ ^]^ and the SST‐driven field‐free magnetization switching measurements,^[^ ^42^ ^]^ where the Néel vector‐dependent and crystal direction‐dependent properties indicate the presence of SST. However, the inverse ASSE is still limited to the spin Seebeck measurement,^[^ ^43^ ^]^ where the thermal effect‐free spin pumping measurement is still waiting to be discovered. Moreover, the comprehensive study of direct and inverse ASSE in the same material systems is important for the consistent understanding of the microscopic mechanism of ASSE.

In this work, we study the direct and inverse ASSE in (101)‐oriented RuO_2_ film with tilted Néel vector ***N***. First, the spin torque along the *x*‐, *y*‐, and *z*‐axis is obtained from the ST‐FMR measurement, and *z*‐axis spin torque is only obvious when the electric current is along the [010] direction. By employing the current‐induced modulation of damping (MOD) method to evaluate *ξ*_DL_ in RuO_2_/Py more directly, the anisotropic ASSE contribution is further confirmed which dominates at lower temperatures. Then, we perform the spin pumping measurement for the study of the inverse ASSE (IASSE) for the (101)‐RuO_2_, where the crystal direction‐dependent spin pumping signals (maximized along the [010] direction) and the enhancement of anisotropic spin pumping signals at lower temperature indicates the IASSE in RuO_2_. This work gives a comprehensive and consistent understanding of the spin splitting effect in altermagnetic RuO_2_.

## 2 Results and Discussion

**Figure 1:**
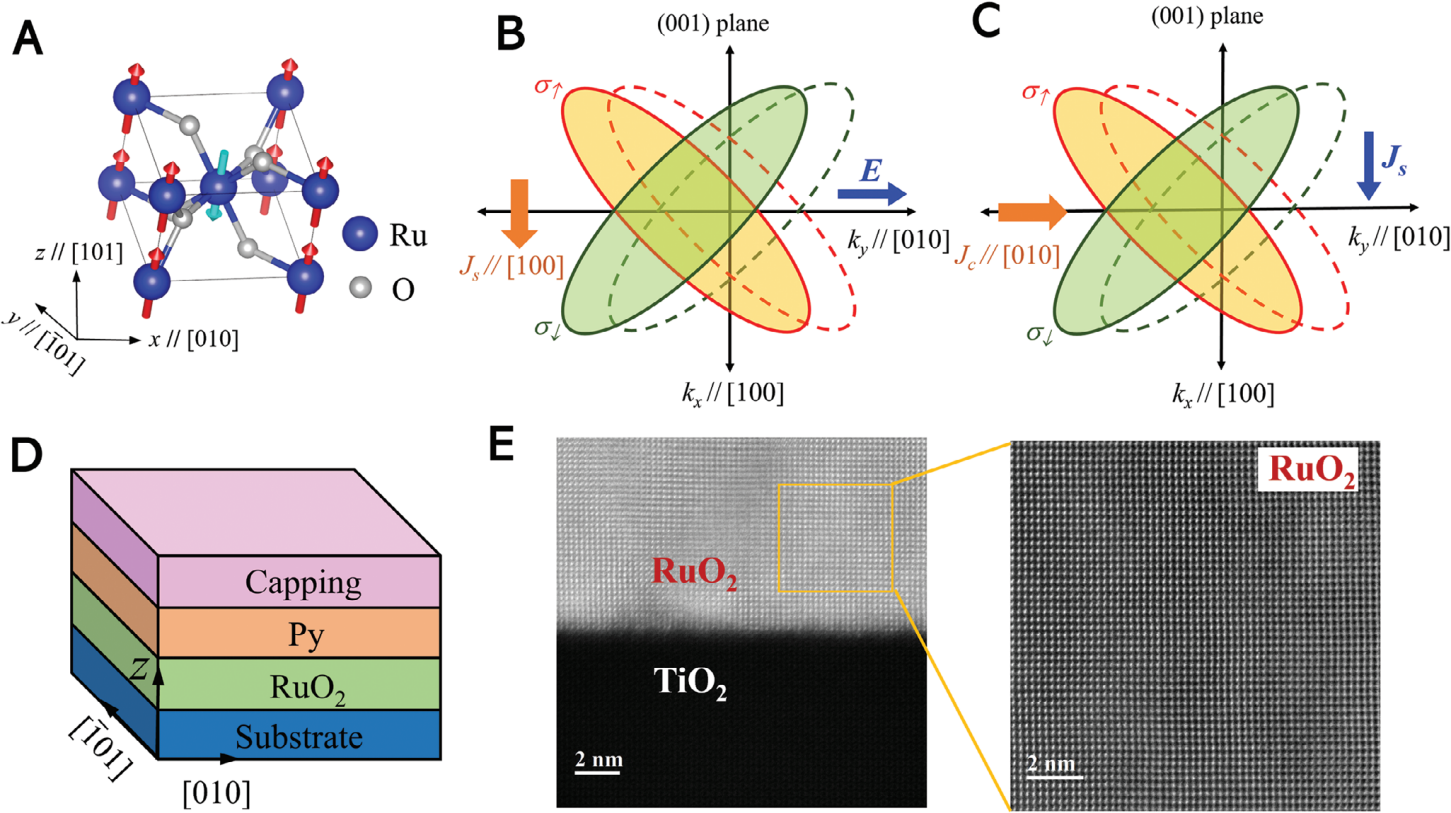
Direct and inverse spin splitting effect in RuO_2_. A) The crystal structure of (101)‐oriented RuO_2_ with the Néel vector ***N*** (arrows) tilted from the out‐of‐plane direction. Blue and grey balls represent Ru and O atoms, respectively. B) Schematic diagrams of charge‐to‐spin and C) spin‐to‐charge conversation via ASSE and IASSE in RuO_2_ from the view of momentum space. D) Schematic structure for the ASSE and IASSE in (101)‐RuO_2_/Py system. E) High‐resolution HAADF image of the cross‐section of TiO_2_//RuO_2_ (left) and RuO_2_ (right) thin film.

RuO_2_ is a conductive rutile oxide, with the Ru atoms at the center and corners of the unit cell having opposite spins, forming a collinear antiferromagnetic ordering with the Néel vector ***N*** oriented along the [001] axis,^[^ ^28^, ^44^ ^]^ as shown in **Figure** **1**A. The center and corners of the Ru atoms experience different oxygen environments, which leads to the anisotropic spin sub‐bands splitting.^[^ ^25^, ^27^ ^]^ As a result of this spin splitting, it can generate a spin current ***J*** *_s_* with the polarization ***σ*** along the ***N*** occurring under an electric field (*E*) (Figure 1B) via ASSE,^[^ ^40^, ^41^, ^42^ ^]^ even without the presence of spin–orbit interaction (SOI), and it is odd under time reversal (T‐odd). Moreover, when introducing a pure spin current polarized parallel to ***N*** into RuO_2_, this spin current induces a non‐equilibrium shift of the Fermi surface, leading to the emergence of a charge current ***J*** *_c_* through its inverse effect called IASSE, as depicted in Figure 1C. This distinctive spin splitting mechanism, along with its inverse effect, contributes significantly to both the generation and detection of spin current.

**Figure 2:**
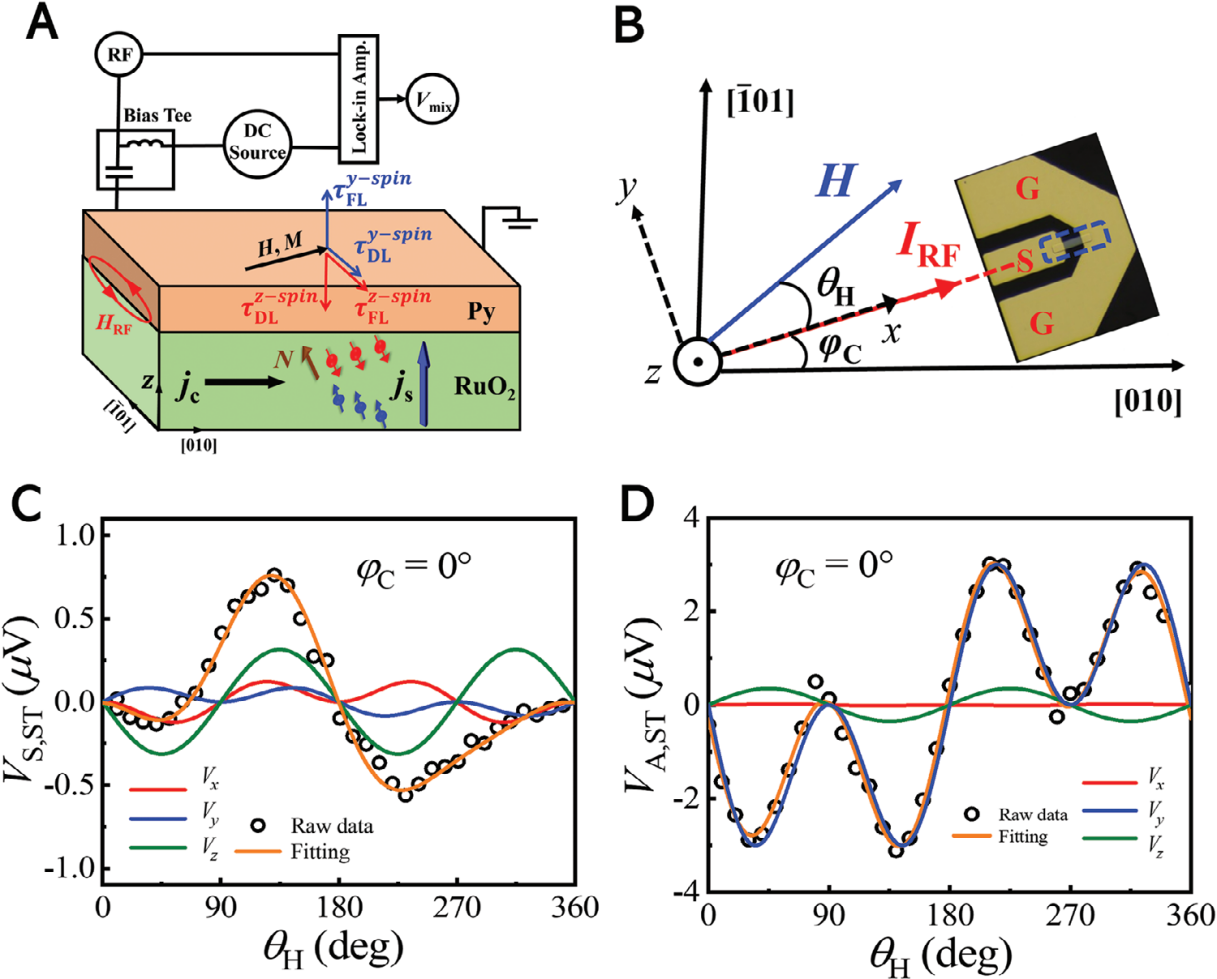
Spin–torque ferromagnetic resonance measurements. A) Schematic of ST‐FMR measurement setup. The *I*_RF_‐induced charge‐to‐spin conversation in RuO_2_ injects a spin current into the Py and excites the FMR. 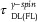 are in‐plane (out‐of‐plane) for *y*‐spin with the blue arrows. 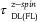 are out‐of‐plane (in‐plane) for *z*‐spin with the red arrows. B) Schematic illustration of the ST‐FMR measurement in (101)‐RuO_2_/Py. The heterostructures are patterned into long stripes within dashed blue rectangles. *θ*_H_ is the angle orientation of the magnetic field *H* relative to the direction of the applied microwave current *I*_RF_ in the sample, and *φ*_C_ is the angle between the applied current and the crystal direction [010] for RuO_2_(101). C) *V*_S,ST_ and D) *V*_A,ST_ voltage amplitudes as a function of *θ*_H_ at *φ*_C_ = 0°. Orange lines represent angle‐dependent voltage signals contributed by the *V_x_* (red), *V_y_* (blue), and *V_z_* (green), based on the analysis by using Equations (2) and (3).

To investigate the ASSE, we fabricate the epitaxially grown RuO_2_ thin films (15 nm) on the TiO_2_(101) single crystalline substrates by magnetron sputtering. And then an in‐plane magnetized Ni_81_Fe_19_(Py) layer (8 nm) with the capping layers of MgO(2 nm)/Ta(3 nm) are deposited, as shown in Figure 1D. High‐resolution cross‐sectional scanning transmission electron microscopy (HRTEM) is conducted in TiO_2_(101)//RuO_2_(101), as shown in Figure 1E, showing the high‐quality epitaxy growth of the RuO_2_(101) film. The high‐resolution high‐angle annular dark field (HADDF) image reveals highly ordered alternatively stacked Ru and O atoms. Next, the devices for ST‐FMR and spin pumping measurements are fabricated by the standard photolithography combined with the ion etching method. First, we use the technique of ST‐FMR^[^ ^45^, ^46^ ^]^ to quantify the SST by ASSE at room temperature. The measurement setup is shown in **Figure** **2**A, including a bias‐tee, a microwave signal generator, and a lock‐in amplifier. An RF current (*I*_RF_) is applied through the sample, which excites the magnetization precession by the ASSE‐induced spin torque. The magnetization resonance contributes to RF resistance due to the anisotropic magnetoresistance (AMR), and the mixing between the RF resistance and the RF current results in a DC voltage signal *V*_mix_, i.e., the spin rectification effect. The geometry of the ST‐FMR device with dashed blue rectangles is illustrated in Figure 2B, which has a dimension of 20 µm in width and 100 µm in length.

The detected ST‐FMR signal *V*_mix_ as a function of the applied in‐plane magnetic field *H* for the (101)‐oriented RuO_2_(15)/Py(8)/MgO(2)/Ta(3) (thicknesses in nanometers) sample is obtained at *θ*_H_ = 40° and *φ*_C_ = 0° (Figure S4, Supporting Information), with a microwave of 18 dBm and 10 GHz. Py film has an ultralow saturation field and isotropic in‐plane magnetic anisotropy, and thus the magnetization is approximately aligned with the external magnetic field *θ*_M_ = *θ*_H_. The *V*_mix_ can be fitted by combining the symmetric (*V*_S,ST_) and antisymmetric (*V*_A,ST_) Lorentzian peak shapes^[^ ^45^, ^46^, ^47^ ^]^:

(1) $$\begin{array}{ccc} & & V_{\mathrm{mix}\;}=\;V_{\mathrm{offset}}\;+\;V_{S,\mathrm{ST}}\frac{\Delta H^{2}}{{\left(H\;-\;H_{r}\right)}^{2}\;+\;\Delta H^{2}}\\ & & \;+\;V_{A,\mathrm{ST}}\frac{\Delta H(H\;-\;H_{r})}{{\left(H\;-\;H_{r}\right)}^{2}\;+\;\Delta H^{2}} \end{array}$$

where *V*_offset_ is the offset, *V*_S,ST_ and *V*_A,ST_ are the coefficients of symmetric and antisymmetric terms, respectively. *H*_r_ is the resonant field and Δ*H* is the linewidth. The angle *θ*_H_ dependence of the *V*_mix_ as an external magnetic field is obtained by rotating the sample plane relative to the magnetic field direction. The amplitude of the damping‐like (DL) and field‐like (FL) torques of *x*‐, *y*‐ and *z*‐polarized spin current can be separated from the *φ*_H_ dependence from the fitting by Equations. (2) and (3):

(2)

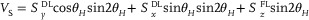



(3)

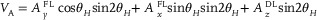

where 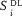 and 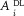 (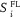 and 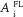) represent the amplitude of the DL (FL) torque generated by the *x*‐, *y*‐ and *z*‐directional spin polarization components, respectively, corresponding to the subscript *i*. Through the analysis by Equations. (2) and (3), the amplitudes of the DL and FL torques are effectively fitted and separated. Figure 2C,D depict the results of *V*_S,ST_ and *V*_A,ST_ measurements at *φ*_C_ = 0°, respectively. It is evident that the observed data cannot be fitted by only considering the conventional spin Hall effect (SHE) ($S_{\;y}^{\;\mathrm{DL}}\cos\varphi \sin2\varphi$) term due to the strict orthogonal relationship among the applied charge current, generated spin current and the spin polarization. The fitting amplitude is derived from the contributions of $\tau_{z}^{\mathrm{FL}}$ and $\tau_{z}^{\mathrm{DL}}$, indicating the emergence of *z*‐spin polarizations. Furthermore, the corresponding ST efficiency, $\xi_{y}^{\mathrm{ST}}\;=\;\frac{S_{y}^{\mathrm{DL}}}{A_{y}^{\mathrm{FL}}}\frac{e\mu_{0}M_{s}t_{\mathrm{Py}}t_{\mathrm{Ru}O_{2}}}{\hbar }\sqrt{1\;+\;\frac{M_{\mathrm{eff}}}{H_{0}}}$ and 
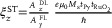
, can be estimated through the extracted amplitudes *V*_S_ and *V*_A_.^[^ ^40^ ^]^ The first term 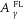 in Equation (3) is predominant from the Oersted field.^[^ ^45^, ^48^, ^49^ ^]^ Here, *e*, *t*_Py_, $t_{\mathrm{Ru}O_{2}}$, *ћ*, and *H*_0_ are the elementary charge, thickness of Py, thickness of RuO_2_, the reduced Planck constant, and Py resonance field at 10 GHz, respectively. 4π*M*_eff_ (9479 Oe) is the effective demagnetization field by fitting the resonance fields by Kittel equation, which is consistent with the saturation magnetization 4π*M*_s_ of 10053 Oe measured by the vibrating sample magnetometry (VSM), see the Supporting Information. The calculated results show that the *z*‐spin torque efficiency for *φ*_C_ = 0° (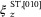
= 0.0155) is stronger than that of *φ*_C_ = 90° (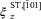
= 0.0037), indicating the crystal direction‐dependent anisotropic ASSE contribution of RuO_2_. The *y*‐spin torque efficiency is 0.137 (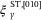) for *φ*_C_ = 0°, and the one for *φ*_C_ = 90° is 0.0166 (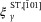), as listed in **Table** **1**. For the (101)‐oriented RuO_2_ film with tilted ***N***, besides the *y*‐polarization, the *z*‐spin polarizations emerge due to the anisotropic spin band splitting of RuO_2_, which agrees with previous experiments^[^ ^40^, ^41^, ^42^ ^]^ and theoretical predictions.^[^ ^22^, ^23^, ^26^ ^]^

**Table 1:** The ST efficiencies and pure spin pumping signal from the [010] and $\left[\bar{1}01\right]$ orientations in (101)‐RuO_2_(15)/Py(8) sample.

Structure	Method	*φ*_C_ = 0° [010] [µV]	*φ*_C_ = 90° $[\bar{1}01]\;$[µV]	Ratio
RuO_2_(15)/Py(8)	ST‐FMR	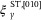	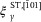	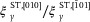
		0.137	0.0166	8.250
	SP	$V_{S,O,\mathrm{SP}}^{\left[010\right]}$	$V_{S,O,\mathrm{SP}}^{\left[\bar{1}01\right]}$	|$V_{S,O,\mathrm{SP}}^{\left[010\right]}$/$V_{S,O,\mathrm{SP}}^{\left[\bar{1}01\right]}$|
		1.85	0.21	8.81

Through the ST‐FMR measurements taken along two orthogonal directions, the obtained ST efficiencies encompass both DL and FL torques. Furthermore, a modulation of damping (MOD) method is performed to further characterize the DL torque efficiency *ξ*_DL_,^[^ ^45^ ^]^ where the Δ*H* of resonance can be tuned by the DC current‐induced SST in the RuO_2_ layer. By varying the DC, the modulation of Δ*H* can be expressed as^[^ ^45^, ^49^ ^]^:

(4) $$\Delta H\;=\;\frac{2\pi f}{\gamma }\;\left[\;+\;\frac{J_{\mathrm{DCDL}}\sin_{H}}{\left(H\;+\;2\pi M_{\mathrm{eff}}\right)\;\mu_{0}M_{0}t_{FM}}\frac{\hbar }{2e}\right]$$

**Figure 3:**
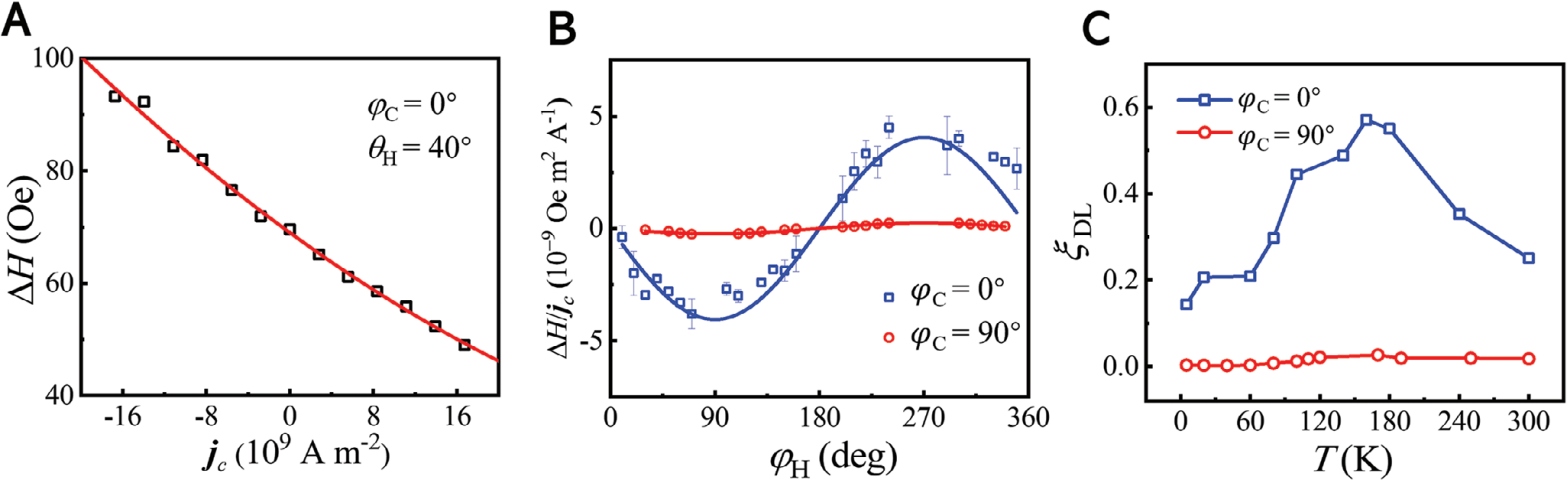
Experimental results for the modulation of damping. A) Δ*H* as a function of current density ***j*** *_c_* in (101)‐RuO_2_(15)/Py(8)/MgO(2)/Ta(3) sample at *θ*_H_ = 40° and the applied current along the *φ*_C_ = 0°. B) The current‐induced modulation of the effective resonance linewidth ∆*H*/***j*** *_c_* as a function of external magnetic field angle *θ*_H_ at *φ*_C_ = 0° (blue square) and *φ*_C_ = 90° (red square), respectively. The solid lines represent the fit to the sin*φ*_H_. C) The extract *ξ*_DL_ as a function of the temperature for (101)‐RuO_2_(15)/Py(8)/MgO(2)/Ta(3) sample at *φ*_C_ = 0° and *φ*_C_ = 90°.

As shown in **Figure** **3**A, the approximately linear relation between Δ*H* and ***j*** *_c_* is extracted. Here, the current is applied along the *φ*_C_ = 0° and the external magnetic field is oriented at the angle *θ*_H_ = 40° with respect to the current direction. The applied frequency and power of microwave are 7 GHz and 18 dBm, respectively. According to Equation (4), we examine the current‐induced change in the effective resonance linewidth ∆*H*/***j*** *_c_* (slope of the linear part fit in Figure 3A) as a function of in‐plane magnetic field *θ*_H_, which fits to sin*θ*_H_. It can be clearly seen that the amplitude of ∆*H*/***j*** *_c_* at *φ*_C_ = 0° (*ξ*_DL,[010]_ = 0.2581) is much stronger than that of *φ*_C_ = 90° (*ξ*_DL,[_ $\bar{1}$_01],_ = 0.0155) at room temperature. The MOD measurement has the advantage of eliminating the spin pumping contribution in ST‐FMR and therefore gives a more accurate value of *ξ*_DL_. Furthermore, the extracted *ξ*_DL_ as a function of the temperature for (101)‐RuO_2_(15)/Py(8)/MgO(2)/Ta(3) sample at *φ*_C_ = 0° and *φ*_C_ = 90° is shown in Figure 3C, from which we observe a strong enhancement of *ξ*_DL_ with decreasing temperature at *φ*_C_ = 0°, indicating a much‐dominated spin splitting contributions at lower temperatures. In contrast, conventional T‐even mechanism resulting from intrinsic SHE generally exhibits negligible temperature dependence when *φ*_C_ = 90°. Hereby, we have demonstrated the generation of the *y*‐ and *z*‐polarized spin current via ASSE in RuO_2_(101). Besides, the DL torque is a strong enhancement with decreasing temperature related to the crystal axes of RuO_2_, provides further evidence to the nontrivial charge‐to‐spin conversation due to the existence of the ASSE in RuO_2_(101), where the decreasing trend below ≈160 K originates from the structural distortion of the TiO_2_ substrate (see the Supporting Information). The observation of temperature‐dependent characteristics of the ASST prompts us to perform its inverse effect in RuO_2_(101)/Py heterostructure.

**Figure 4:**
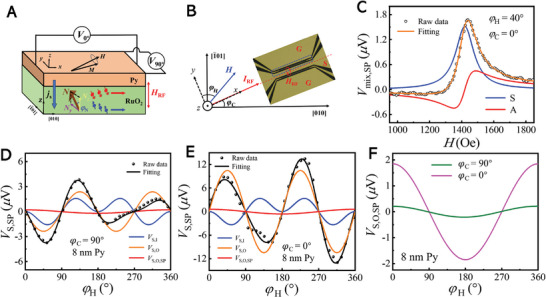
Angular dependence of DC voltages for the spin pumping measurement. A) The schematic diagram of the spin pumping measurement for device configuration with an out‐of‐plane microwave excitation field *H*_RF_ in (101)‐RuO_2_/Py. *φ*_N_ is the angle between the ***N*** and the *z*‐axis. B) The actual depiction of the spin pumping device configuration with an out‐of‐plane microwave excitation field *H*_RF_ in (101)‐RuO_2_/Py. *φ*_H_ is the angle of the applied magnetic field *H* relative to the *y*‐axis and the layout includes two orthogonal directional components *V*_0°_ and *V*_90°_. C) The DC voltage signals (*V*_mix,SP_) obtained for (101)‐RuO_2_(15)/Py(8)/MgO(3)/Ta(3) sample at *φ*_H_ = 40° and the spin pumping device layout along the *φ*_C_ = 0°, where the fitting results include symmetric and antisymmetric parts. Symmetric voltage amplitudes (*V*_S,SP_) as a function of angle *φ*_H_ for D) *φ*_C_ = 90° and E) *φ*_C_ = 0°, respectively. Black plots and curves show the raw data and fitting parameters, contributed by the *V*_S,I_ (blue), *V*_S,O_ (yellow), and *V*_S,O,SP_ (red), fitting by the Equations. (5) and (6). F) The extracted pure spin pumping voltage signals (*V*_S,O,SP_) as a function of *φ*_H_.

Next, the IASSE is studied by the spin pumping measurement in the stack of (101)‐RuO_2_(15)/Py(8)/MgO(3)/Ta(3), as shown in **Figure** **4**A. The spin‐pumping device configuration has a dimension of 8 µm in width and 360 µm in length. The stripe located in the gap of the coplanar waveguide (CPW) experiences an out‐of‐plane microwave excitation field via the ground–source–ground (GSG) pads, which has the advantage of the separation of spin pumping and parasitic anisotropic magnetoresistance,^[^ ^50^ ^]^ as shown in Figure 4B. Here, the spin‐pumping device utilizes the same thin film as the ST‐FMR device in Figure 2, to enable a comparison between the direct and inverse spin‐splitting effects in RuO_2_. Figure 4C shows a typical spin pumping signal of *V*_mix,SP_‐*H* curve (offset voltage *V*_offset_ is subtracted) under the microwave power *P* = 23 dBm, an excitation frequency of 10 GHz, *φ*_H_ = 40,° and *φ*_C_ = 0° at room temperature. The symmetric (blue line) and antisymmetric Lorentzian (red line) curves are employed to describe the line–shapes of the spectra, and the *V*_mix,SP_ is fitted well by the sum of *V*_S,SP_ *L*_S,SP_ and *V*_A,SP_ *L*_A,SP_, where *V*_S,SP_ and *V*_A,SP_ are the magnitudes of symmetric and antisymmetric components, respectively. After considering the finite inductive currents induced in the conductive RuO_2_ layer which can lead to an additional in‐plane magnetic excitation field, the DC voltages as a function of *φ*_H_ are fitted by the following equations:

(5) $$V_{A,SP}=V_{A,I}\sin^{2}\varphi_{H}\cos\varphi_{H}+V_{A,O}\sin2\varphi_{H}$$

(6) $$V_{S,SP}=V_{S,I}\sin^{2}\varphi_{H}\cos\varphi_{H}+V_{S,O}\sin2\varphi_{H}+V_{S,O,SP}\cos\varphi_{H}$$

Here, *V*_A,I_ (*V*_A,O_) and *V*_S,I_ (*V*_S,O_) are the coefficients of *V*_A,SP_ and *V*_S,SP_ induced by the in‐plane (out‐of‐plane) excitation,^[^ ^51^ ^]^ and *V*_S,O,SP_ is the magnitude of the spin pumping signal (SP) induced by the out‐of‐plane excitation (see the Supporting Information). The *φ*_H_ represents the angle of the applied magnetic field *H* relative to the *y*‐axis. The antisymmetric part in Figure 4C solely arises for the AMR‐spin rectification as described in Equation (5). However, the amplitude of sthe ymmetric part contains pure SP (*V*_S,O,SP_) and AMR‐spin rectification parts. The *V*_S,SP_ signal as a function of *φ*_H_ is extracted from the Lorentzian line–shape to acquire the *V*_S,O,SP_ by Equation (6), as shown in Figure 4D,E, corresponding to *φ*_C_ = 90° and 0°, respectively. For the pure SP contribution, the obtained value of $V_{S,O,\mathrm{SP}}^{\;[\bar{1}01]}$ is 0.21 µV in Figure 4D with *φ*_C_ = 90° owing to the conventional spin‐to‐charge (*σ_y_*_(_ *_x_*_)_) conversion with the *x*‐axis spin polarization, without the spin polarization component from ***N*** in *yz*‐plane (the schematic diagram of Figure 4A). Note that the ***J*** *_s_* is along the *z*‐axis and the detected DC voltage *V*_90°_ is along the *y*‐axis. In contrast, the *V*_S,O,SP_ in the case with *φ*_C_ = 0° is increased to 1.85 µV ($V_{S,O,\mathrm{SP}}^{\;[010]}$) in Figure 4E when *V*_0°_ is detected along the *x*‐axis. Here, the spin polarization in the *y*‐axis (*σ_y_*) includes the conventional *σ_y_*_(_ *_y_*_)_ and ***N***‐dependent *σ* ***_N_*** *_y_*_(_ *_y_*_)_, indicating that the enhanced charge current arises from the IASSE‐induced spin‐to‐charge conversation in the RuO_2_ layer. Subsequently, Figure 4F shows the comparison of the pure SP contributions with the voltage signal along the orthogonality directions (*φ*_C_ = 90° and *φ*_C_ = 0°) obtained from the fitting of Figure 4D,E, and a substantial enhancement of *V*_S,O,SP_ is observed at *φ*_C_ = 0° compared to *φ*_C_ = 90°, where this difference exhibits the features of IASSE with the Néel vector‐dependent spin‐to‐charge conversion, which is also consistent with theoretical prediction^[^ ^25^ ^]^ and experimental results.^[^ ^43^ ^]^ As presented in Table 1, a comparison of both spin pumping and ST‐FMR for the (101)‐RuO_2_(15)/Py(8)/MgO(3)/Ta(3) film at *φ*_C_ = 90° [$\bar{1}$01] and *φ*_C_ = 0° [010], it reveals that the anisotropy in the spin‐pumping signal (|$V_{S,O,\mathrm{SP}}^{\;[010]}$/$V_{S,O,\mathrm{SP}}^{\;[\bar{1}01]}$| = 8.25) is consistent with the anisotropy observed in the ST‐FMR signals (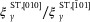 = 8.81). The direct and inverse spin splitting effects, as well as the crystal direction‐dependent anisotropic behavior for the samples, confirming the spin splitting effects in the (101)‐RuO_2_.

Table 1 summarizes the ST efficiencies and pure spin pumping signal in (101)‐RuO_2_ for the two orthogonal directions *φ*_C_ = 0° ([010]) and *φ*_C_ = 90° ([$\bar{1}01$]).

**Figure 5:**
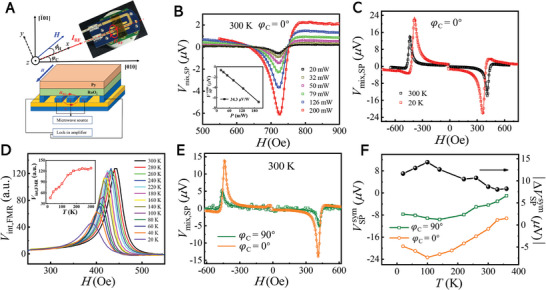
Temperature dependence of spin pumping signal. A) The actual illustration (top) and schematic diagram (bottom) of the spin pumping measurement with an in‐plane *H*_RF_ in a 5 × 5 mm^2^ dimension of (101)‐RuO_2_(15)/Py(20)/MgO(3)/Ta(3) film, attached at the top of the CPW. *ϕ*_H_ is the orientation of the applied magnetic field *H* relative to *I*_RF_. B) The resulting DC voltages acquired with microwave power *P* ranging from 13 to 23 dBm at *φ*_C_ = 0°. The inset illustrates the *P*‐dependence of magnitudes for the symmetric ($V_{\mathrm{SP}}^{\;\mathrm{sym}}$, black squares) components of the DC voltages. C) The dependence of voltage signal on magnetic field at typical temperatures *T* of 20 K (red) and 300 K (black). D) Integral FMR spectra for different *T*, the inset shows the dependence of the FMR amplitude on *T*. E) Comparison of magnetic field‐dependent voltage signals for *φ*_C_ = 0° (yellow) and *φ*_C_ = 90° (green) orientations, at 300 K. F) *T*‐dependent behavior of the $V_{\mathrm{SP}}^{\;\mathrm{sym}}$ signal for *φ*_C_ = 0° (yellow) and *φ*_C_ = 90° (green) orientations, on the left axis. The right axis shows the temperature dependence of the $V_{\mathrm{SP}}^{\;\mathrm{sym}}$ difference between these two orientations, i.e., |Δ$V_{\mathrm{SP}}^{\;\mathrm{sym}}$|−*T* (black). The above measurements are taken with *ϕ*_H_ = 0° where the magnetic field is parallel to the *I*_RF_.

To obtain the temperature dependence of IASSE, we perform the in‐plane spin‐pumping measurement in the (101)‐RuO_2_(15)/Py(20)/MgO(3)/Ta(3) film where a 20 nm Py film is used to ensure a sufficiently strong signal in FMR measurement, by attaching the film to a CPW holder which is integrated inside the cryogenic chamber of the physical property measurement system (PPMS). **Figure** **5**A illustrates the actual illustration (top) and schematic diagram (bottom) of the spin pumping measurement. The (101)‐RuO_2_(15)/Py(20)/MgO(3)/Ta(3) film is positioned on top of the CPW and has tight winding around it, causing the microwave field to align in‐plane. The detected voltage, orthogonal to *I*_RF_, is measured by attaching two electrodes at the ends of a 4.5 mm distance to quantify the induced voltages. Initially at room temperature, the (101)‐RuO_2_(15)/Py(20)/MgO(3)/Ta(3) film is measured with *φ*_H_ = 0° and *φ*_C_ = 0°, at 6 GHz and varied *P* ranging from 13 to 23 dBm, as shown in Figure 5B. The measured symmetric component is represented by *V*_sym_ (*ϕ*_H_) = $V_{\mathrm{SP}}^{\;\mathrm{sym}}$ cos*ϕ*_H_ + $V_{\mathrm{AMR}}^{\;\mathrm{sym}}$ sin2*ϕ*_H_sin*ϕ*_H_, where $V_{\mathrm{SP}}^{\;\mathrm{sym}}$ and $V_{\mathrm{AMR}}^{\;\mathrm{sym}}$ denote the amplitudes of the pure SP and AMR‐spin rectification contributions to the symmetric component, respectively. Notably, the AMR‐spin rectification contribution becomes null at *ϕ*_H_ = 0° for a pure Py layer, resulting in a symmetric signal for the RuO_2_/Py sample solely attributed to SP. Hence, we concentrate on the *ϕ*_H_ = 0° configuration with the magnetic field being parallel to the *I*_RF_. In Figure 5B, we extract the symmetric component ($V_{\mathrm{SP}}^{\;\mathrm{sym}}$) for SP analysis, where the slight asymmetric signal might be from the induced RF driving current through the sample.^[^ ^50^, ^52^ ^]^ The inset of Figure 5B shows the $V_{\mathrm{SP}}^{\;\mathrm{sym}}$ as a function of *P*, with a linear relation.

Then, we carry out the spin pumping measurement with *ϕ*_H_ = 0° at different temperatures. The typical temperatures of 20 K (red) and 300 K (black) are displayed in Figure 5C, showing the *H*‐dependent voltage signals at *P* = 23 dBm, where the reversed polarity of *V*_mix,SP_ at positive and negative magnetic fields indicating the evidence of spin pumping effect. The extracted $V_{\mathrm{SP}}^{\;\mathrm{sym}}$ the component at varied *T* is summarized in Figure 5F (yellow circles). Notably, as the *T* decreases from 300 to 100 K, even the FMR intensity reduces at lower temperatures as shown in Figure 5D,^[^ ^53^ ^]^ the $V_{\mathrm{SP}}^{\;\mathrm{sym}}\;$ gradually increases and reaches a double value compared to that at 300 K, as shown in Figure 5F, where the transition below 100 K comes from the crystal phase transition of the TiO_2_ substrate.^[^ ^54^ ^]^ Further to clarify the detailed relationship between the crystal phase transition of the TiO₂ substrate and the altermagnetic spin splitting effect, the Raman spectroscopy measurements and first‐principles calculations of the spin splitting energy in the band of RuO_2_ are performed (see the Supporting Information). Generally, the spin splitting of the band structure is much dominated at lower temperatures due to the suppression of the thermal fluctuations, which is consistent with the enhanced $V_{\mathrm{SP}}^{\;\mathrm{sym}}\;$ by IASSE in RuO_2_ with reducing the temperature to 100 K.

The same method is performed for the detection voltage along another direction *φ*_C_ = 90°. In Figure 5E, the *V*_mix,SP_ signal for *φ*_C_ = 0° is 2.9 times larger than that for *φ*_C_ = 90°, confirming the crystal orientation‐dependent IASSE of RuO_2_. Furthermore, both *φ*_C_ = 0° and *φ*_C_ = 90° exhibits a consistent trend as varying *T* ranging from 10 to 300 K, and $V_{\mathrm{SP}}^{\;\mathrm{mix}}$ signal for *φ*_C_ = 0 is significantly stronger than that of *φ*_C_ = 90° at all range of temperature. The difference (|Δ$V_{\mathrm{SP}}^{\;\mathrm{sym}}$|) between these two orientations is much more significant at lower temperatures (8.7 µV at 300 K and 14.2 µV at 100 K), indicating the much‐dominated anisotropic spin splitting effect at a lower temperature. Furthermore, as the temperature increases, the spin‐pumping signal gradually decreases. At 390 K, the signal‐noise ratio is very low, and fitting with the Lorentzian curve is not feasible (see Supporting Information).

In the altermagnetic RuO_2_, the spin polarization direction is dependent on the Néel vector of RuO_2_ due to the spin splitting effect. The crystal structure of (101)‐oriented RuO_2_ with the Néel vector tilted from the out‐of‐plane direction in *y*–*z* plane determines the spin polarization direction (*y‐* and *z*‐spin). We performed a comprehensive analysis of anisotropy in both ST‐FMR and SP signals using a RuO_2_/Py(8 nm) sample. Simultaneous measurements are conducted for both ST‐FMR and spin pumping devices on this film. The observed anisotropy in the spin‐pumping signal aligns consistently with the anisotropy observed in the ST‐FMR signals. The presence of direct and inverse spin‐splitting effects, along with the crystal direction‐dependent anisotropic behavior in the samples, provides confirmation of the spin‐splitting effects in the (101)‐RuO_2_ orientation.

The analysis of the differences between the spin torque efficiency (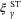 by the angle‐dependent ST‐FMR) and the DL torque efficiency (*ξ*_DL_ by current‐modulated damping) has been discussed and refined. For *φ*_C_ = 90° ([$\bar{1}$01] orientation), the ASSE can be ignored, thus one can analyze the ST‐FMR data by the method of general heavy metals. The spin torque efficiency quantified by angle‐dependent ST‐FMR contains the contribution of FL torque and DL torque. The relationship of 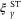, *ξ*_DL_, and *ξ*_FL_ are written as 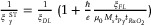.Obviously, 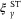 is not usually equal to *ξ*_DL_ if *ξ*_FL_ cannot be ignored. For the Py/RuO_2_ bilayer, the difference between 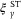 and *ξ*_DL_ is quite significant, compared with traditional heavy metals, which may be caused by the conductivity evaluation for the RuO_2_ layer. We estimate the current distribution in Py/RuO_2_ by using the conductivity values that are measured in separated Py and RuO_2_ films. As we know, *ξ*_DL_ calculated by current‐modulated damping needs an accurate current density inside the RuO_2_ layer in the Py/RuO_2_ heterostructure. However, the diffusion of oxygen and the interfacial electric field at the interface between the Py and RuO_2_ layer changes the distribution of charge carriers near the interface and thus affect the conductivity inside the RuO_2_ layer, which leads to the difficulty of the accurate current density calculations inside the RuO_2_ layer in the Py/RuO_2_ heterostructure. We put most of our efforts into the crystal dependence for one specific measurement to analyze the properties of ASSE in the RuO_2_ layer. Nevertheless, the differences in measured data between different methods are also very interesting and need to be further studied in the future.

## 3 Conclusion

In summary, we have studied the direct and inverse spin splitting effect in (101)‐oriented altermagnetic RuO_2_, by the ST‐FMR, current‐induced modulation of damping and spin pumping measurements. The direct ASSE is detected by the ST‐FMR and MOD methods: the spin currents along the *z*‐direction arising from the ASSE show an anisotropic behavior and are maximized when an electric current is along the [010] direction; MOD gives consistent anisotropic ASSE result, where the ASSE is enhanced at lower temperatures due to much‐dominated spin splitting of the band structure. The spin pumping method is employed to detect the spin current by the IASSE in (101)‐orientation RuO_2_, and crystal direction‐dependent spin pumping signals demonstrate the IASSE in RuO_2_ which is along the [010] direction. Furthermore, the pronounced temperature‐dependent spin pumping signals and the $V_{\mathrm{SP}}^{\;\mathrm{sym}}$ difference between two crystal directions ([010] and [$\bar{1}$01]) further confirm a dominated spin splitting in RuO_2_ at lower temperatures. These results give a consistent understanding of the direct and inverse spin splitting effect in altermagnetic RuO_2_, with great potential for future altermagnetic materials and devices with flexibly controlled spin polarization.

## 4 Experimental Section

### Sample Growth and Characterizations

The RuO_2_/Ni_81_Fe_19_(Py)/MgO/Ta film was gown on the TiO_2_ (101) substrate by magnetron sputtering in a system maintained at a base pressure of 3 × 10^−8^ Torr. During the deposition, the Ar atmosphere was maintained with a pressure of 2.5 mTorr. The RuO_2_ layer was deposited by radio frequency (RF) sputtering. The Py and Ta layers were deposited by direct current (DC) sputtering and the sputtering power was 30 W. The MgO layer was deposited by RF sputtering with 80 W.

The thickness and crystal structure were characterized by X‐ray reflection (XRR) and X‐ray diffraction (XRD) techniques with a Bruker D8 Discover diffractometer using Cu K_α_ radiation (λ = 0.15419 nm). By using the technique of photolithography and ion etching, the multilayer films were patterned into standard rectangular‐shaped strips with 20 × 100 µm2 for ST‐FMR, 8 × 360 µm2 for spin pumping measurements, and 20 × 100 µm2 for longitudinal resistivity, respectively. Cr(10 nm)/Au(100 nm) metal stacks were deposited as coplanar waveguide (CPW) transmission lines or contacts for electrical measurements.

The magnetic properties of the samples were measured by the vibrating sample magnetometer (VSM) module of a superconducting quantum interface device (SQUID).

### Characteristics and Methods of Magnetic Dynamics

For ST‐FMR measurement, a microwave produced by a microwave signal generator was applied to the device. The RF power ranged from 13 to 23 dBm, and the frequency was ranging from 3 to 10 GHz. A mixing DC voltage would be measured by a lock‐in amplifier. A bias‐tee was used to divide RF and DC signals.

For spin‐pumping measurements, the devices were placed in a vacuum chamber with a base pressure of 2×10^−4^ mbar and at varied temperatures from 10 to 300 K. The microwave current with a frequency ranging from 3 to 10 GHz was used and the input microwave power ranging from 13 to 23 dBm. A microwave probe was attached to the G–S–G electrode to produce an RF magnetic field (*H*_RF_) around the signal line. The heterostructure devices were placed at the gap between the signal and ground line. A DC voltage was measured by a lock‐in amplifier.

For the spin pumping measurement of films, the film was tightly wound on the CPW by Kapton tape. The FMR measurement system, designed by NanoSC company, was used to obtain voltage signals. In this setup, two electrons were attached to the ends of the distance of 4.5 mm to acquire the induced voltages. The spin‐pumping measurement was performed in a physical property measurement system (PPMS) at various temperatures from 10 to 300 K.

## Conflict of Interest

The authors declare no conflict of interest.

## Supporting information

Supporting Information

## Data Availability

The data that support the findings of this study are available in the supplementary material of this article.
