# Supplementary material for: Direct and Inverse Spin Splitting Effects in Altermagnetic RuO2
Source: Adv Sci (Weinh). 2024 Apr 16;11(25):2400967. doi: 10.1002/advs.202400967 (PMC11220717; doi:10.1002/advs.202400967)
Supplement: Supplementary file 1 — Supporting Information [file ADVS-11-2400967-s001.pdf]

ADVANCED SCIENCE
Open Access

# Supporting Information

for *Adv. Sci.*, DOI 10.1002/advs.202400967

Direct and Inverse Spin Splitting Effects in Altermagnetic $RuO_2$

*Yaqin Guo, Jing Zhang, Zengtai Zhu, Yuan-yuan Jiang, Longxing Jiang, Chuangwen Wu, Jing Dong, Xing Xu, Wenqing He, Bin He, Zhiheng Huang, Luojun Du, Guangyu Zhang, Kehui Wu, Xiufeng Han, Ding-fu Shao, Guoqiang Yu* and Hao Wu**

Supplementary Materials for

# Direct and Inverse Spin Splitting Effects in Altermagnetic $RuO_2$


Yaqin Guo[1†], Jing Zhang[1†], Zengtai Zhu[1†], Yuan-yuan Jiang[2,3], Longxing Jiang[1], Chuangwen Wu[1], Jing Dong[1], Xing Xu[1], Wenqing He[4], Bin He[4], Zhiheng Huang[4], Luojun Du[1,4], Guangyu Zhang[1,4], Kehui Wu[1,4], Xiufeng Han[1,4], Ding-fu Shao[2,3], Guoqiang Yu[1,4*], and Hao Wu[1,4*]

*[1]Songshan Lake Materials Laboratory, Dongguan, Guangdong 523808, China*

*[2]Key Laboratory of Materials Physics, Institute of Solid State Physics, HFIPS, Chinese Academy of Sciences, Hefei 230031, China*

*[3]University of Science and Technology of China, Hefei 230026, China*

*[4]Beijing National Laboratory for Condensed Matter Physics, Institute of Physics, Chinese Academy of Sciences, Beijing 100190, China*

[†]These authors contributed equally to this work.

[*]To whom correspondence should be addressed: guoqiangyu@iphy.ac.cn (Guoqiang Yu); wuhao1@sslab.org.cn (Hao Wu)

**This PDF file includes:**

Supplementary Text: Supplementary 1 to 11

Figs. S1 to S13

References

**Supplementary 1**: Sample structural characterization and surface morphology.

**Supplementary 2**: In-plane magnetic measurement for the $RuO_2$/Py sample.

**Supplementary 3**: Longitudinal resistivity of $RuO_2$(101).

**Supplementary 4**: The results of ST-FMR of the (101)-$RuO_2$/Py sample prepared using the magnetron sputtering and the PLD method.

**Supplementary 5**: Methods for angle-dependent DC voltages in (101)-$RuO_2$/Py sample for the out-of-plane excitation employed.

**Supplementary 6**: The IASSE with crystal direction-dependent anisotropic behavior in(101)-$RuO_2$/Py(20) sample for the out-of-plane excitation employed.

**Supplementary 7**: Temperature dependence of spin pumping signal in (101)-$RuO_2$/Py(20) sample.

**Supplementary 8**: The temperature dependence of the IASSE above 300K.

**Supplementary 9**: The measured FMR spectra at typical temperature.

**Supplementary 10**: The detailed configuration of the actual device.

**Supplementary 11**: Raman spectrum of $TiO_2$//$RuO_2$ at different temperatures and first-principles calculations.

## Supplementary 1. Sample structural characterization and surface morphology.

We deposited 20 nm-thick $RuO_2$ into a single crystalline $TiO_2(101)$ substrate. The film was analyzed by using the X-ray diffraction (XRD), exhibiting the (101) and (202) peaks as shown in Fig. S1(a). The deposition speed was determined via x-ray reflectivity (XRR), depicted Fig. S1(b). Additionally, the atomic force microscopy (AFM) image illustrates the surface morphology of $RuO_2$ thin film in Fig. S1(c). The topographical can be observed by various parameters that exist to quantify the root mean square (rms) roughness of a surface. The RMS roughness of $RuO_2$ thin film is 0.158 nm, indicating a flat surface of the $RuO_2$ film.

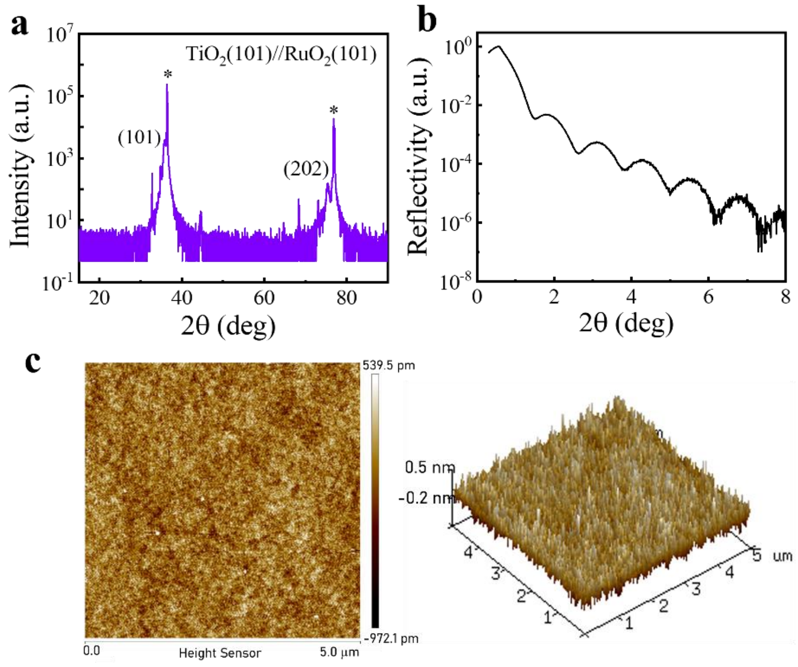


**Fig. S1.** (a) XRD spectra and (b) XRR curve of 20 nm-thick $RuO_2(20)$ film grown on a $TiO_2(101)$ substrate. (c) AFM images of $RuO_2$ thin film.

## Supplementary 2. In-plane magnetic measurement for the $RuO_2$/Py sample.

The in-plane magnetic hysteresis loop for the $TiO_2(101)//RuO_2(15)/Py(20)/MgO(3)/Ta(3)$ sample was measured by the vibrating sample magnetometer (VSM) module of a superconducting quantum interface device (SQUID). The loop exhibits a saturation magnetization value of 800 meu/cc for the Py

layer.

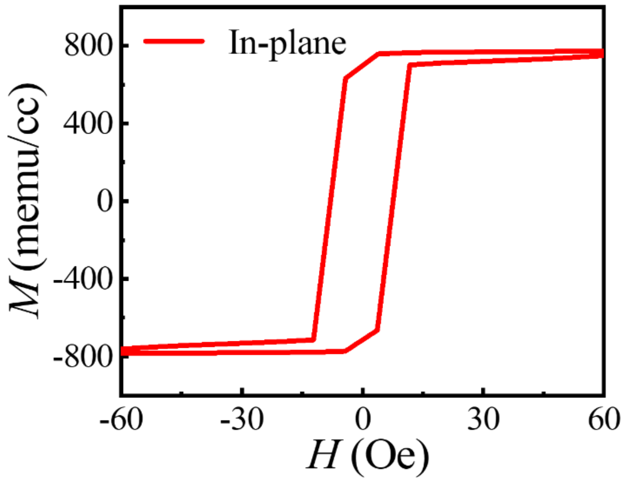


**Fig. S2**. The in-plane magnetic fields of the $TiO_2$(101)//$RuO_2$(15)/Py(20)/MgO(3)/Ta(3) sample.

**Supplementary 3. Longitudinal resistivity of $RuO_2$(101).**

Fig. S3 shows the dependence results on the crystal angle $\varphi_C$. The resistivity $\rho_{xx}$ is ~ 130 $\mu\Omega$ cm for 15 nm thick (101)-$RuO_2$ film at room temperature.

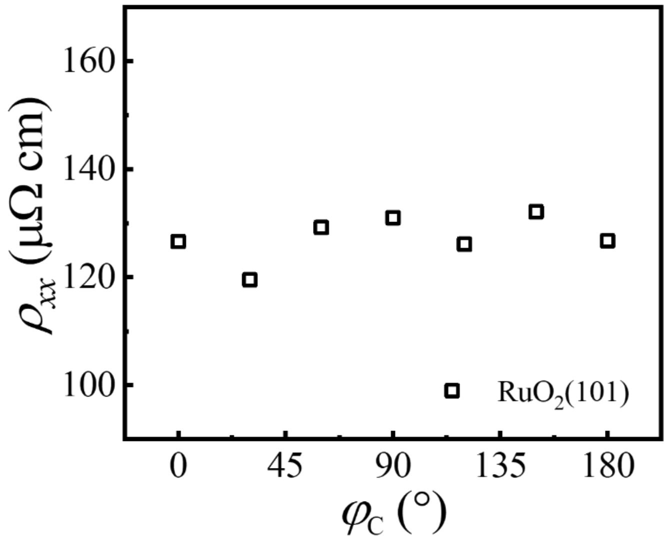


**Fig. S3**. Longitudinal resistivities $\rho_{xx}$ for (101)-$RuO_2$ as a function of the crystal angle $\varphi_C$.

## Supplementary 4.

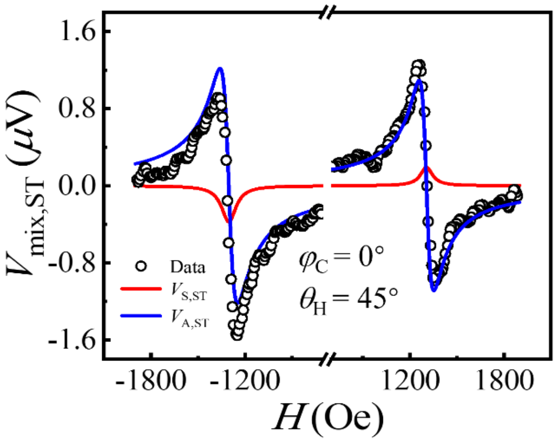


**Fig. S4.** The mixing ST-FMR voltage of the (101)-$RuO_2$(15)/Py(8)/MgO(2)/Ta(3) (thicknesses in nanometers) sample prepared using the magnetron sputtering method at $\theta_H$ = 45°and $\varphi_C$ = 0° as a function of applied in-plane magnetic field. Black circles show the raw data, which are fitted by symmetric ($V_{S,ST}$, red curve) and antisymmetric ($V_{A,ST}$, blue curve) components.

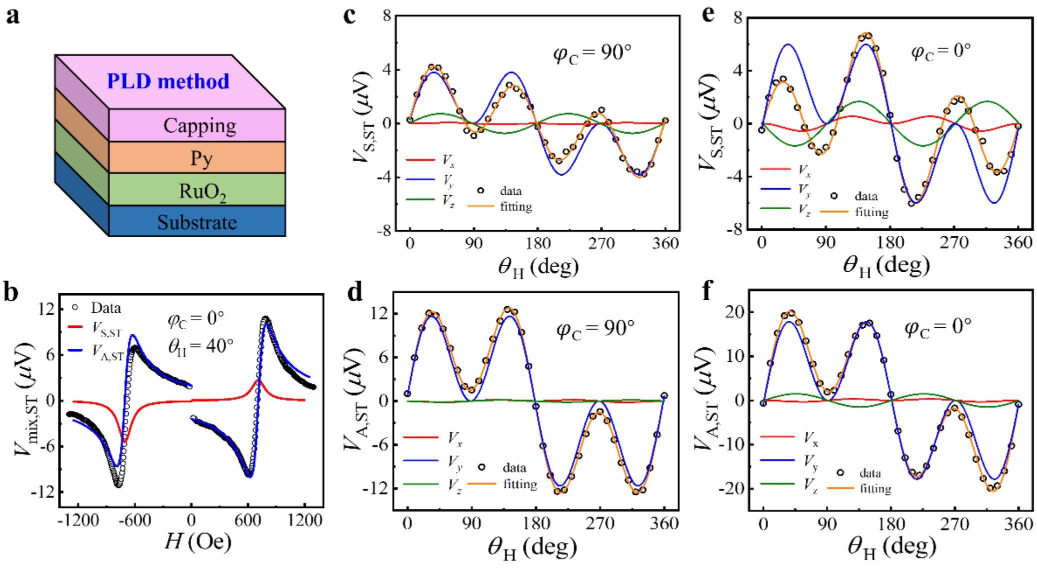


**Fig. S5. Angular dependence of ST-FMR signals.** (a) Schematic diagram of the structure for (101)-$RuO_2$/Py prepared using the PLD method. (b) The mixing voltage of the (101)-$RuO_2$(15)/Py(8)/MgO(2)/Ta(3) (thicknesses in nanometers) sample at $\theta_H$ = 40°and $\varphi_C$ = 0° as a function of applied in-plane magnetic field. Black circles show the raw data, which are fitted by

symmetric ($V_{S,ST}$, red curve) and antisymmetric ($V_{A,ST}$, blue curve) components. (b) $V_{S,ST}$ and (c) $V_{A,ST}$ voltage amplitudes as a function of $\theta_H$ at $\varphi_C = 90°$. (e) $V_{S,ST}$ and (f) $V_{A,ST}$ voltage amplitudes at $\varphi_C = 0°$. Orange lines represent angle-dependent voltage signals contributed by the $V_x$ (red), $V_y$ (blue), and $V_z$ (green), based on the analysis by using the Eqs. (2) and (3).

Fig. S5 shows the ST-FMR measurement for (101)-$RuO_2$/Py prepared using the PLD method. Fig. S5(b) shows the detected ST-FMR signal $V_{mix,ST}$ as a function of the applied in-plane magnetic field $H$ for the (101)-oriented $RuO_2$(15)/Py(8)/MgO(2)/Ta(3) (thicknesses in nanometers) sample at $\theta_H = 40°$and $\varphi_C = 0°$, with a microwave of 18 dBm and 7 GHz. Figs. S5(c) and S5(d) depict the results of $V_S$ and $V_A$ measurements at $\varphi_C = 90°$, respectively. The obvious $y$-spin polarization ($\tau_y^{DL}$) $\propto$ $\cos\theta_H \sin 2\theta_H$ from the conventional spin Hall effect (SHE) is due to the strict orthogonal relationship among the applied charge current, generated spin current and the spin polarization. The value of $|S_x^{DL}/S_y^{DL}|$ and $|S_z^{FL}/S_y^{DL}|$ is 0.015 and 0.14, respectively, indicating that the major contribution from $\sigma_y$. However, for the applied current along the [010] direction with $\varphi_C = 0°$, the corresponding data in Figs. S5(e) and S5(f) cannot be fitted by only considering the $S_y^{DL}\cos\varphi\sin 2\varphi$ term. By fitting the Eq. (2), the enhanced amplitude is originated from $\tau_x^{DL}$ and $\tau_z^{FL}$, and $|S_x^{DL}/S_y^{DL}|$ and $|S_z^{FL}/S_y^{DL}|$ is 0.12 and 0.22, respectively, revealing that $x$-, $z$-spin polarizations emerge.

**Supplementary 5**

The $V_{ISHE}$ resulting from the spin pumping is directly proportional to the cross product of the spin current $\boldsymbol{J}_s$ (parallel to the z-axis) and the spin polarization vector $\boldsymbol{\sigma}$ (parallel to the $\boldsymbol{M}$). In spin pumping experiments, the generation of $\boldsymbol{J}_s$ is derived from momentum transfer of magnetization procession. Meanwhile, the component of dynamic magnetization (mainly contain $m_y$ and $m_z$) also be affected. One can estimate $V_{ISHE}$ by dynamic magnetization. Therefore, $V_{ISHE}$ can be expressed as follow:

$$V_{ISHE} \sim (\boldsymbol{J}_s \times \boldsymbol{\sigma})_{x'} \sim [\mathrm{Im}(m_y)\mathrm{Re}(m_z)\text{-}\mathrm{Re}(m_y)\mathrm{Im}(m_z)]\cos\varphi_H \quad (1)$$

Where Re($m_y$), Im($m_y$), Re($m_z$) and Im($m_z$) are the real and imaginary parts of $m_y$ and $m_z$. $\varphi_H$ is the angle of the applied magnetic field $H$ relative to the $y$-axis [Fig. 4(b)]. In the measurement configuration, the rectification voltage aligns with the inductive microwave current in Py $\boldsymbol{j}_{Py}$(t), which can be expressed as

$$V_{AMR} \sim \boldsymbol{j}_{Py}\mathrm{Re}(m_y)\sin 2\varphi_H \tag{2}$$

The dynamic magnetization is linked to the dynamic magnetic susceptibility and driving fields [1, 2], which can be written as

$$\begin{pmatrix} m_y \\ m_z \end{pmatrix} = \begin{pmatrix} \chi^{\mathrm{I}} & -i\chi_{\mathrm{a}}^{\mathrm{O}} \\ i\chi_{\mathrm{a}}^{\mathrm{I}} & \chi^{\mathrm{O}} \end{pmatrix} \begin{pmatrix} h_{\mathrm{Oe}}^{\mathrm{RuO_2}} \sin\varphi \, e^{\mathrm{i}\phi_1} \\ h^{\mathrm{O}} e^{\mathrm{i}\phi_2} \end{pmatrix} \tag{3}$$

Where $\chi^{\mathrm{I}}$ ($\chi_{\mathrm{a}}^{\mathrm{I}}$) represents the complex diagonal (off-diagonal) dynamic magnetic susceptibility attributed to in-plane excitation, $\chi^{\mathrm{O}}$ ($\chi_{\mathrm{a}}^{\mathrm{O}}$) is the complex diagonal (off-diagonal) dynamic magnetic susceptibility associated with out-of-plane excitation, and $\phi_1$ ($\phi_2$) is the phase shift between the dynamic magnetization and $h_{\mathrm{Oe}}^{\mathrm{RuO_2}}$ ( $h^{\mathrm{O}}$ ). Applicable to the out-of-plane excitation used in Fig. 4 in the main text.

As $h_{\mathrm{Oe}}^{\mathrm{RuO_2}}$ is Oersted field induced by the secondary microwave current $\boldsymbol{j}_{Py}$(t) in Py layer, while $h^{\mathrm{O}}$ is space magnetic field directly induced by the CPW, $\phi_1$ and $\phi_2$ is usually not equal. It should be noted that each component of the susceptibility $\chi$ has both real and imaginary parts, $\chi$ = Re($\chi$) + iIm($\chi$). Thus, based on Supplementary Equation 1-3, the angular dependence of $V_{ISHE}$ and $V_{AMR}$ under in-plane ($h_{\mathrm{Oe}}^{\mathrm{RuO_2}}$) and out-of-plane ($h^{\mathrm{O}}$) excitations can be derived as

$$V_{ISHE}^{I} \sim [h_{\mathrm{Oe}}^{\mathrm{RuO_2}}]^2 sin^2\varphi_H \, cos\varphi_H \tag{4}$$

$$V_{AMR}^{I} \sim j_{Py}\, h_{\mathrm{Oe}}^{\mathrm{RuO_2}} sin2\varphi_H \, sin\varphi_H \sim j_{Py} h_{\mathrm{Oe}}^{\mathrm{RuO_2}} sin^2\varphi_H \, cos\varphi_H \tag{5}$$

$$V_{ISHE}^{O} \sim [h^{\mathrm{O}}]^2 cos\varphi_H \tag{6}$$

$$V_{AMR}^{O} \sim j_{Py} h^{\mathrm{O}} \, sin2\varphi_H \tag{7}$$

where, $V_{ISHE}^{I}$ and $V_{AMR}^{I}$ are in-plane ISHE voltage and AMR rectification voltage, respectively; $V_{ISHE}^{O}$ and $V_{AMR}^{O}$ are out-of-plane ISHE voltage and AMR rectification voltage, respectively.

For the out-of-plane excitation employed, it is possible to distinguish the symmetric components of $V_{\mathrm{ISHE}}$ and AMR due to the distinct angular dependencies revealed in supplementary Eq. (6) and (7). Notably, $V_{\mathrm{ISHE}}$ reaches maximum at $\varphi_{\mathrm{H}} = 0°$ (M $\perp$ the stripes), while all other signals are equal to zero. This geometric configuration provides us with the opportunity to accurately measure pure spin pumping signals in the $RuO_2$/Py system. Therefore, the expressions for $V_{\mathrm{A,SP}}$ and $V_{\mathrm{S,SP}}$ [Eqs. (5) and (6) in the main text] are derived to fit the experimentally measured DC voltage. Theses equations perfectly explain the experimental data, explicitly demonstrating the angular dependence of the DC voltage resulting from both in-plane and out-of-plane excitations.

**Supplementary 6**

The IASSE is studied by the spin pumping measurement in the stack of (101)-$RuO_2$(15)/Py(20)/MgO(3)/Ta(3), where a 20 nm Py film is used to ensure a sufficiently strong signal in FMR measurement, as shown in Fig. S6a. The $V_{\mathrm{S,SP}}$ signal as a function of $\varphi_{\mathrm{H}}$ is extracted from the Lorentzian line-shape to acquire the $V_{\mathrm{S,O,SP}}$ by Eq. (6), as shown in Figs S6(c) and S6(d), corresponding to $\varphi_{\mathrm{C}} = 90°$ and 0°, respectively. For the pure SP contribution, the obtained value of $V_{\mathrm{S,O,SP}}$ is 0.56 $\mu$V in Fig. S6c with $\varphi_{\mathrm{C}} = 90°$ owing to the conventional spin-to-charge ($\sigma_{y(x)}$) conversion with the $x$-axis spin polarization, without the spin polarization component from $\boldsymbol{N}$ in $yz$-plane (the schematic diagram of Fig. S6(a). Note that the $\boldsymbol{J}_s$ is along $z$-axis and the detected DC voltage $V_{90°}$ is along the $y$-axis. In contrast, the $V_{\mathrm{S,O,SP}}$ in the case with $\varphi_{\mathrm{C}} = 0°$ is increased to 1.07$\mu$V in Fig. S6d when $V_{0°}$ is detected along the $x$-axis. Here, the spin polarization in $y$-axis ($\sigma_y$) includes the conventional $\sigma_{y(y)}$ and $\boldsymbol{N}$-dependent $\sigma_{\boldsymbol{N}y(y)}$, indicating that the enhanced charge current arises from the IASSE-induced spin-to-charge conversation in $RuO_2$ layer.

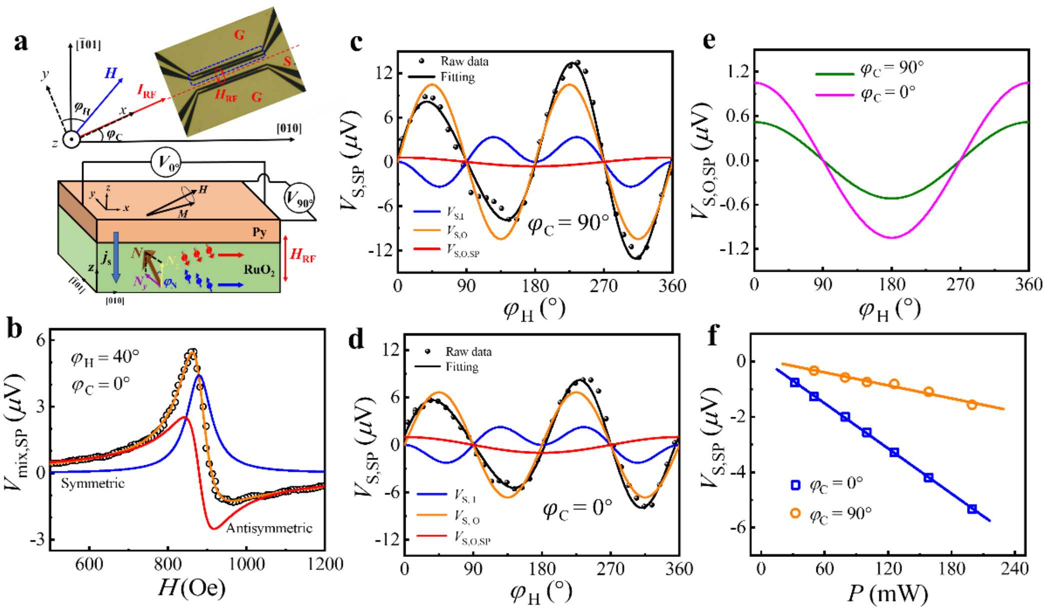


**Fig. S6. Angular dependence of DC voltages for the spin pumping measurement.** (a) The actual depiction (top) and schematic diagram (bottom) of the spin pumping measurement for device configuration with an out-of-plane microwave excitation field $H_{RF}$ in (101)-$RuO_2$/Py. $\varphi_H$ is the angle of the applied magnetic field $H$ relative to the $y$-axis and the layout includes two orthogonal directional components $V_{0^\circ}$ and $V_{90^\circ}$. $\varphi_N$ is angle between the $\boldsymbol{N}$ and the $z$-axis. (b) The DC voltage signals ($V_{mix,SP}$) obtained for (101)-$RuO_2$(15)/Py(20)/MgO(3)/Ta(3) sample at $\varphi_H = 40°$ and the spin pumping device layout along the $\varphi_C$ = 0°, where the fitting results include symmetric and antisymmetric parts. Symmetric voltage amplitudes ($V_{S,SP}$) as a function of angle $\varphi_H$ for (c) $\varphi_C = 90°$ and (d) $\varphi_C = 0°$, respectively. Black plots and curves show the raw data and fitting parameters, contributed by the $V_{S,I}$ (blue), $V_{S,O}$ (yellow), and $V_{S,O,SP}$ (red), fitting by the Eqs. (5) and (6). (e) The extracted pure spin pumping voltage signals ($V_{S,O,SP}$) as a function of $\varphi_H$. (f) Microwave power dependence of the extracted symmetric voltage component ($V_{S,SP}$) for both $\varphi_C = 0°$ and $\varphi_C = 90°$ orientations.

Different from the samples with 8 nm Py above, here, the 20 nm Py is used. And the SP signal decreases relative to the AMR-spin rectification signal, due to the increase of Py layer thickness aligning with the experimental expectations. Indeed, one can see that the crystal orientation-dependent IASSE of $RuO_2$ confirms the feature of IASSE with the Néel vector-dependent spin-to-charge conversion. The result validates the reliability of our data. Furthermore, the voltage signal with $\varphi_H = 0°$ is predominantly

attributed to the contributions of pure SP-induced through IASSE or ISHE instead of AMR-spin rectification contribution, based on Eq. (6). Hence, the pure SP signal was measured with the device orientation close to $\varphi_H = 0°$ (Fig. S6), a summary of the $V_{S,SP}$ values acquired at different $P$ when the voltage was detected along [010] ($\varphi_C = 0°$) and [$\bar{1}$01] ($\varphi_C = 90°$) directions. Evidently, linear relationships are apparent between $V_{S,SP}$ and $P$, and the slope allows for a semiquantitative assessment of the spin-to-charge conversion efficiency. It is worth noting that the amplitude of $V_{S,SP}$ at $\varphi_C = 0°$ (27.2 $\mu$V/W) is more than three times larger than $V_{S,SP}$ at $\varphi_C = 0°$ (7.8 $\mu$V/W), indicating the crystal direction-dependent IASSE besides conventional SHE, which is consistent with the anisotropic spin splitting in the band structure of $RuO_2$ [3, 4].

## Supplementary 7

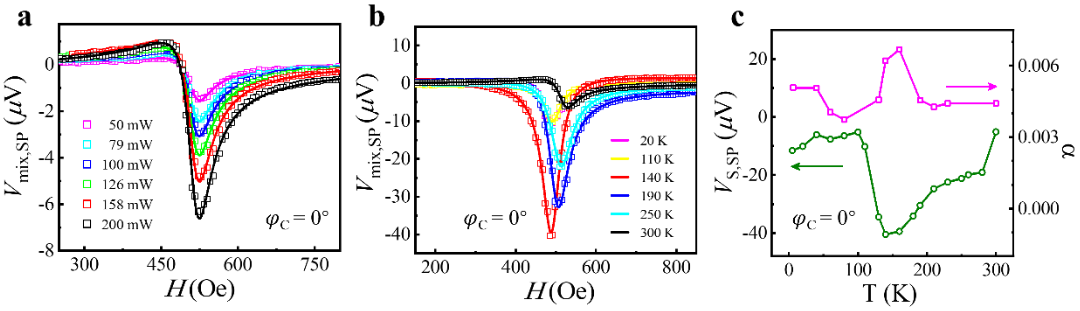


**Fig. S7.** (a) Voltage signals recorded from the (101)-$RuO_2$(15)/Py(20)/MgO(3)/Ta(3) sample for $\varphi_C = 0°$ at 8 GHz and 300 K under different microwave power ranging from 17 to 23 dBm. (b) The obtained voltage signals at different temperature with 23 dBm RF excitation for $\varphi_C = 0°$. (c) Temperature-dependence behavior of the $V_{S,SP}$ signal (left axis) and damping (right axis). These measurements are taken with the device positioned close to $\varphi_H = 90°$.

We focus on the pure SP signal measured with the device positioned close to $\varphi_H = 90°$. We delve into the measurements conducted at varying temperatures. Initially, measurement is carried out at room temperature by using the same device of (101)-$RuO_2$(15)/Py(20)/MgO(3)/Ta(3) stack. As shown in Fig. S7(a), the measurements are performed with $\varphi_C = 0°$ at different microwave power ($P$) ranging from 17 to 23 dBm, at 8 GHz and 300 K. The observed slight asymmetric signal might be arisen the

inductive or capacitively coupled microwave. Taking this into account, we consider the $V_{S,SP}$ signal as the SP signal. In the same device, we carried out measurements of the SP-induced DC voltage as a function of temperature ranging from 20 to 300 K, as depicted in Fig. S7(b). As the temperature decreases, the $V_{mix,SP}$ signal gradually increases at $\varphi_C = 0°$. Similarly, the extracted $V_{S,SP}$ signal from $V_{mix,SP}$ also displays an enhancement as the decreasing the temperature towards 140 K, indicating a more prominent IASSE effect at lower temperature in Fig. S7(c). At the same time, the Gilbert damping coefficient $\alpha$ gradually increases with reducing the temperature, as shown in Fig. S7(c), indicating a much stronger spin pumping efficiency, which is consistent with the enhancement of $V_{S,SP}$ to reflect the magnitude of spin pumping.

In the scenario of $\varphi_C = 90°$, as shown in Fig. S8, a similar trend of $V_{S,SP}$ and $\alpha$ with $T$ variation is demonstrated. The pronounced temperature-dependent spin-pumping signal observed in $RuO_2$ stands in contrast to that seen in heavy metals or oxides.

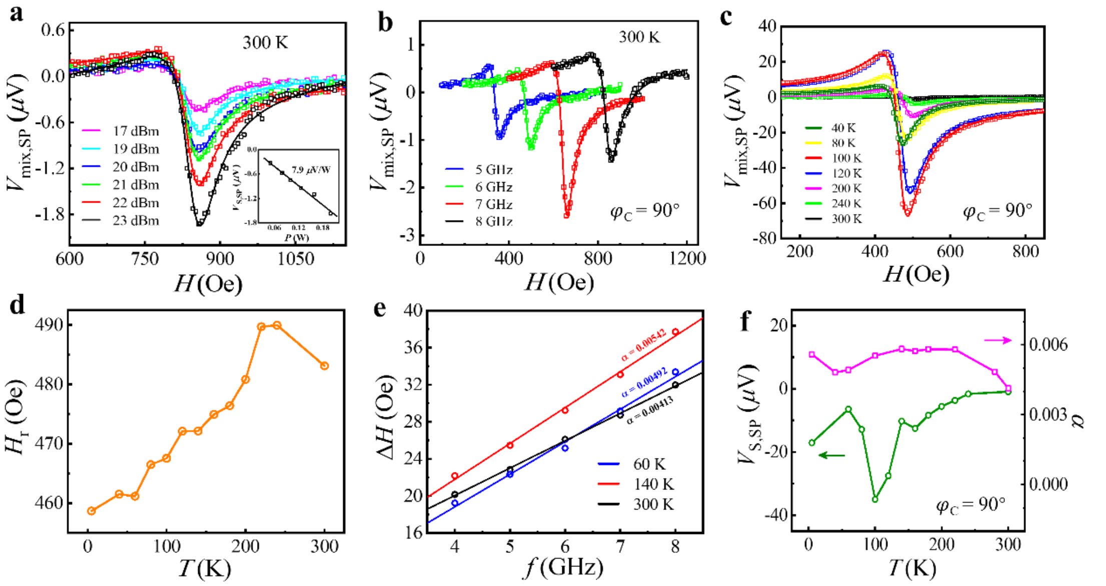


**Fig. S8.** (a) Voltage signals recorded from the $RuO_2$(15)/Py(20)/MgO(3)/Ta(3) sample for $\varphi_C = 90°$ at 8 GHz and 300 K under different microwave power levels ranging from 17 to 23 dBm. (b) Voltage signals acquired from the $RuO_2$(15)/Py(20)/MgO(3)/Ta(3) sample across a range of RF frequencies from 4 to 8 GHz, under microwave power of 23 dBm, at 300 K. (c) Temperature-dependent of $V_{mix,SP}$ under 23 dBm RF excitation for $\varphi_C = 90°$. (d) A summary of the $H_r$ values corresponding to different temperature in (c). (e) A representative plot of $\Delta H$-$f$ at various temperatures and the corresponding

damping $\alpha$. (f) Temperature-dependence behavior of the $V_{S,SP}$ signal and damping. These measurements are taken with the device positioned close to $\varphi_H = 90°$.

## Supplementary 8

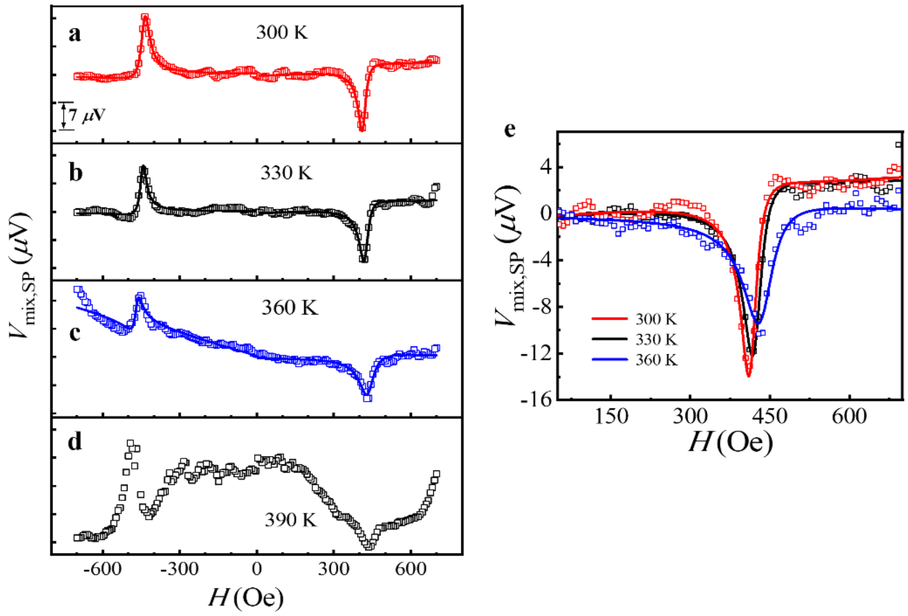


**Fig. S9.** The dependence of spin pumping signal on magnetic field for $\varphi_C = 0°$ orientations of (101)-$RuO_2$(15)/Py(20)/MgO(3)/Ta(3) film at different temperatures $T$, (a) 300 K, (b) 330 K, (c) 360 K and (d) 390 K. (e) The comparison of spin pumping signal for $\varphi_C = 0°$ orientations at 330 K, 330 K and 360 K.

We carry out the spin pumping measurement of the (101)-$RuO_2$(15)/Py(20)/MgO(3)/Ta(3) film with $\varphi_C = 0°$, at 6 GHz and 23 dBm, with higher temperatures 330 K, 360 K and 390 K. Due to the maximum temperature limit of the PPMS equipment (400 K), we measured up to 390 K. As shown in Fig. S9(a)-(d), the reversed polarity of $V_{mix,SP}$ at positive and negative magnetic fields indicating the evidence of spin pumping effect. And as the temperature increases, the spin pumping signal gradually decreases, as shown in Fig. S9(e). At 390 K, the signal-noise ratio is very low, and fitting with the Lorentzian curve is not feasible.

Besides, our measured spin Hall conductivity (SHC) is approximately 20 times

smaller than the prediction for a single-domain $RuO_2$ sample. The device size used is 20 × 100 μm$^2$ for ST-FMR. In such devices, which are multi-domains, and the net contribution from the preferred orientation of the domain distribution can lead to non-zero values of $\xi^E_{DL,[010]}$. Here, $\xi^E_{DL,i}$ is the DL efficiencies per unit applied electric field for each component of torque $i$, $\xi^E_{DL,i} = \xi_{DL,i}/\rho_{xx}$. $\xi^E_{DL,[010]} = 19.8 \times 10^4$ (Ω m)$^{-1}$, $\xi^E_{DL,,[\bar{1}01]} = 1.19 \times 10^4$ (Ω m)$^{-1}$, which is consistent with the previously reported[5]. For a single-domain $RuO_2$, it is predicted that $\sigma^Z_{ZX}$ is more than an order of magnitude larger than $\sigma^Y_{ZX}$. In the future, the nanoscale devices approaching single domain limit will significantly improve the $\sigma^Z_{ZX}$ resulting from the spin splitting effect, as predicted by A. Bose *et al.*[6]

**Supplementary 9**

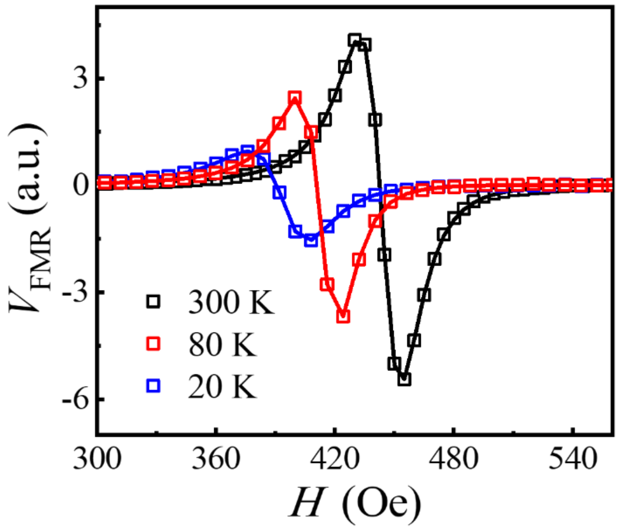


**Fig. S10**. The measured FMR spectra at select typical temperature.

The (101)-$RuO_2$(15)/Py(20)/MgO(3)/Ta(3) film is performed by the FMR at select typical temperature in Fig. S10. FMR testing system is integrated in the PPMS. Notably, as the $T$ decreases from 300 K to 100 K, the FMR intensity reduces at lower temperature.

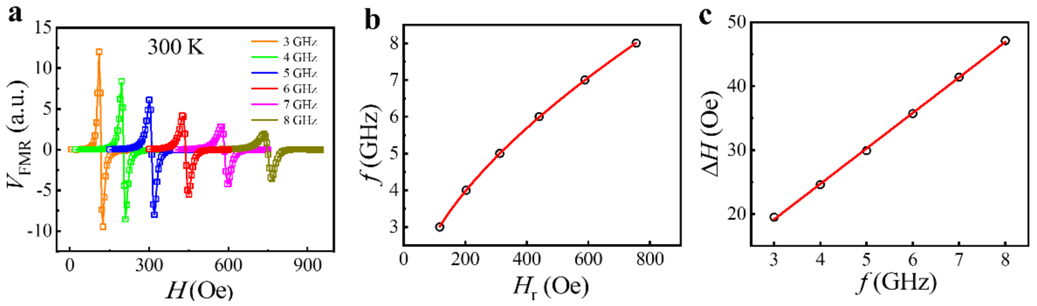


**Fig. S11.** (a) FMR spectra at different microwave frequencies. (b) $H_r$ dependence of the microwave frequency. (c) FMR linewidth as a function of microwave frequency. The dots are measured data, and the lines are fitting results.

The FMR spectra shown in Fig. S11(a) is obtained by FMR measurement system integrated on PPMS. The microwave signal was transformed to a DC voltage signal at the measurement terminal. 20 nm Py film is used to ensure a sufficiently strong signal in FMR measurement. The direct measurement results are Lorentz derivatives because of magnetic field modulation from a Helmholtz coil installed around the sample. $V_{FMR}$ represents the measured voltage signals. Fig. S11(b) shows resonance field dependence of the microwave frequency. According to well-known Kittel equation for in-plane ferromagnetic films:

$$f = \frac{\gamma}{2\pi}\sqrt{(4\pi M_{eff} + H)H} \tag{S1}$$

in which $4\pi M_{eff}$ is effective demagnetization, $H$ is magnetic field. The fitting coefficients shows $4\pi M_{eff}$ = 9479 Oe, $\gamma/2\pi$ = 28 GHz/T, which are agree with the properties of Py monolayer. Fig. S11(c) shows the FMR linewidth as a function of microwave frequency. The dots are measured data, and the lines are fitting results. The Gilbert damping can be obtained by the fitting formula:

$$\Delta H = \Delta H_0 + 2\alpha\omega/\gamma \tag{S2}$$

where, $\Delta H$ is FMR linewidth, $\Delta H_0$ is the inhomogeneous linewidth, which is independent of microwave frequency, $\omega = 2\pi f$ is microwave angle frequency, $\gamma$ is the gyromagnetic ratio and $\alpha$ is the dimensionless Gilbert damping constant. The fitting coefficients show $\Delta H_0$ = 2.527 Oe and $\alpha$ = 0.00777.

**Supplementary 10**

The detailed configuration of the actual device is presented and summarized in Fig. S12.

Fig. S12(a) presents the schematic illustration of the ST-FMR measurement in (101)-$RuO_2$/Py, where the heterostructures are patterned into long stripes within dashed blue rectangles. The geometry of ST-FMR device has a dimension of 20 $\mu$m in width and 100 $\mu$m in length. $\theta_H$ is the angle orientation of the magnetic field relative to the direction of the applied microwave current $I_{RF}$ in the sample, and $\varphi_C$ is the angle between the applied current and the crystal direction [010] for $RuO_2$(101).

In Fig. S12(b), the schematic illustration depicts the spin pumping measurement for device configuration with an out-of-plane microwave excitation field $H_{RF}$ in (101)-$RuO_2$/Py. This spin pumping device configuration has a dimension of 8 $\mu$m in width and 360 $\mu$m in length. The stripe located in the gap of the coplanar waveguide (CPW) experiences an out-of-plane microwave excitation field via the Ground-Source-Ground (GSG) pads.

Furthermore, Fig. S12(c) shows the schematic of the spin pumping measurement for film with an in-plane $H_{RF}$ in (101)-$RuO_2$/Py which is integrated into the PPMS. The spin pumping measurement with an in-plane $H_{RF}$ in a 5 × 5 mm$^2$ dimension of (101)-$RuO_2$/Py film, attached at the top of the CPW. $\phi_H$ is the orientation of the applied magnetic field $H$ relative to $I_{RF}$.

Left side: the actual illustration, and right side: schematic diagram.

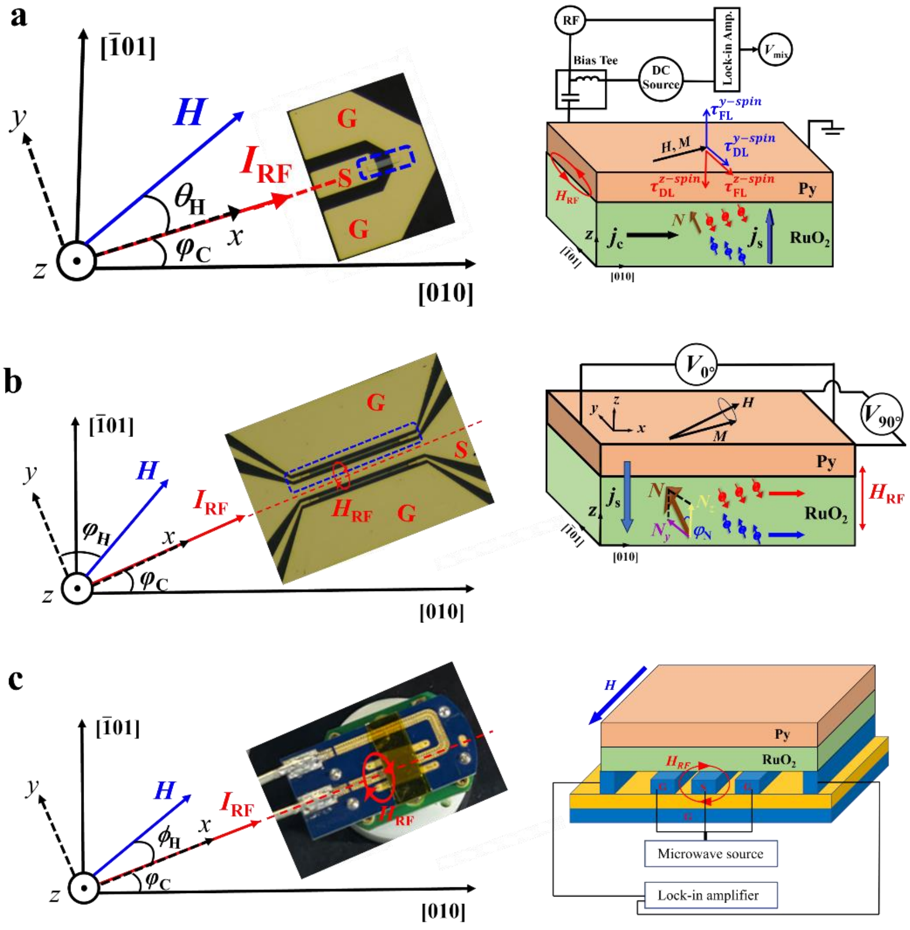

**Fig. S12**. (a) Schematic illustration of the ST-FMR measurement in (101)-$RuO_2$/Py. The heterostructures are patterned into long stripes within dashed blue rectangles. (b) Schematic illustration of the spin pumping measurement for device configuration with an out-of-plane microwave excitation field $H_{RF}$ in (101)-$RuO_2$/Py. (c) The schematic of the spin pumping measurement for film with an in-plane $H_{RF}$ in (101)-$RuO_2$/Py.

It is noteworthy that the Eqs. (5) and (6) in the main text are applied to the out-of-plane excitation, aiming to distinguish the symmetric components of $V_{S,O,SP}$ and AMR due to the distinct angular dependencies revealed. The $V_{S,O,SP}$ reaches maximum at $\varphi_H = 0°$ (M ⊥ the stripes, $V_{S,SP} = V_{S,O,SP} \cos\varphi_H$), with no contribution from the AMR signal, as evident from the Fig. 4. However, the configuration in Fig. 5 is specifically designed for in-plane excitation and it is utilized to further validate the inverse spin splitting effect in $RuO_2$. And the AMR-spin rectification contribution also becomes zero at $\phi_H = 0°$ when the applied magnetic field is parallel to the $I_{RF}$. It is noted that spin pumping

effect only contribute to the symmetric component of measured voltage, while the asymmetric component still exists for $\phi_H = 0°$, as shown in Fig. 5.

**Supplementary 11**

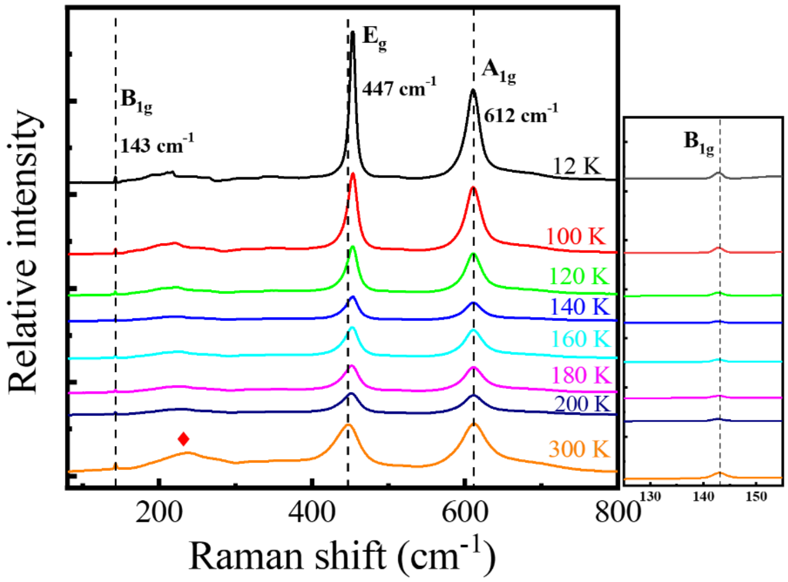


**Fig. S13.** Raman spectrum of $TiO_2$//$RuO_2$(15 nm) at different temperatures.

For the transition of $\xi_{DL}$ and $V_{SP}^{sym}$ at low temperature, we have performed Raman spectroscopy measurements at different temperatures with a micro-Raman spectrometer (Horiba LabRAM HR Evolution). A solid-state laser at 532 nm and below 100 $\mu$W is focused onto the samples along the $z$ direction by a × 100 objective with a spot size less than 1 $\mu$m. The spectrum is collected at different temperatures for $TiO_2$//$RuO_2$(15 nm), as shown in Fig. S13.

Each cell of $TiO_2$ with rutile structure contains two $TiO_2$ molecules, which belong to the P42/mnm space group and Raman vibration is $A_{1g} + B_{1g} + E_g$. The vibration of the 143 cm$^{-1}$ peak $B_{1g}$ is weak, the vibration of the 447 cm$^{-1}$ peak $E_g$ and the vibration of the 612 cm$^{-1}$ peak $A_{1g}$ is strong, which are the characteristic peaks of rutile $TiO_2$//$RuO_2$ [7, 8]. As for the wide spectral band at 239 cm, Hara *et al* [9] believe that it is induced by the large lattice disorder of rutile, and it may also be caused by multi-level scattering or

distortion. The position of the 612 $cm^{-1}$ peak $A_{1g}$ and the 143 $cm^{-1}$ peak $B_{1g}$ show no change basically, while the 447 $cm^{-1}$ peak $E_g$ appears as a distinct blue shift. From the point of view of the peak intensity of the three peaks, the peak intensity gradually decreases with the temperature decreasing from 300 K to 140 K, and reaches the weakest point at the critical temperature with 140 K. Then, with the further decrease of temperature, the peak intensity increases. The above results show that there is structural distortion or energy band change at the range of 140 K to 120 K, which leads to the change of Raman scattering peak intensity, which can further explain the change of $\xi_{DL}$ and $V_{SP}^{sym}$ value with temperature.

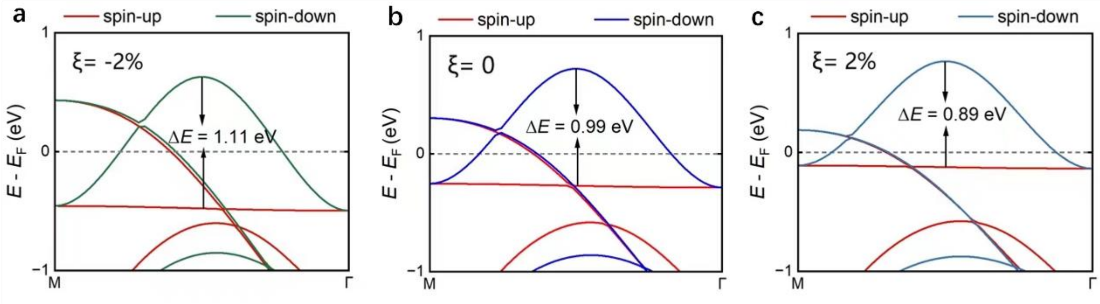


**Fig. S14.** The energy of spin splitting in the band of $RuO_2$ under (a) compressive strain ξ = -2%, (b) no strain ξ = 0 and (c) tensile strain ξ = 2%.

Further to clarify the detailed relationship between the crystal phase transition of the $TiO_2$ substrate and the altermagnetic spin splitting effect, first-principles calculations of the spin splitting energy in band of $RuO_2$ is performed. First-principles calculations were performed based on density functional theory (DFT)[10] as implemented in the Vienna *ab initio* simulation package (VASP).[11, 12] The pseudopotentials were described using the projector augmented wave (PAW) method,[13] and the exchange-correlation functional was treated within the generalized gradient approximation (GGA) developed by Perdew-Burke-Ernzerhof (PBE).[14] In the calculations for $RuO_2$, the cutoff energy for the plane-wave expansion was set to 500 eV, and the k-point grid was set to 16 × 16 × 16 to sample the irreducible Brillouin zone. Besides, The GGA+U[15, 16] method with $U_{eff}$ = 2 eV on Ru 4*d* orbitals was employed. The lattice parameters and the atomic coordinates were relaxed until the force on each atom was less than 0.001 eV/Å for the calculation of the band structure.

We have conducted calculations with a ±2% expansion/compression of the optimized lattice to investigate the influence of the unit cell volume on the spin splitting

in band structure of $RuO_2$. As depicted in Fig. S14(a), with a lattice compression of $\xi$ = -2%, the energy of spin splitting in the band ($\Delta E$) is 1.11 eV, indicating a larger band splitting compared to the case of $\xi$ = 0 (0.99 eV) [Fig. S14(b)], suggesting an increase in SST relative to the $\xi$ = 0 case. However, for a lattice expansion of $\xi$ = 2% [Fig. S14(c)], the $\Delta E$ decreases to 0.89 eV with the SST effect decreases. This is consistent with the results obtained from our experiments. As the temperature decreases from 300 K to 100 K, the $TiO_2$ substrate induces a compressive strain on $RuO_2$, causing an increase in its spin-splitting energy band, and the damping-like torque efficiency and IASSE of $RuO_2$ are increase. At temperatures below 100 K, the $TiO_2$ substrate induces tensile strain on $RuO_2$, leading to a decrease in its spin-splitting energy band and a reduction in the damping-like torque efficiency and IASSE.

## REFERENCES AND NOTES


[1] L. Chen, F. Matsukura, H. Ohno, Nat Commun **2013**, 4, 2055.

[2] L. Chen, S. Mankovsky, S. Wimmer, M.A.W. Schoen, H.S. Körner, M. Kronseder, D. Schuh, D. Bougeard, H. Ebert, D. Weiss, C.H. Back, Nature Phys. **2018**, 14, 490-494.

[3] R. Gonzalez-Hernandez, L. Smejkal, K. Vyborny, Y. Yahagi, J. Sinova, T. Jungwirth, J. Zelezny, Phys. Rev. Lett. **2021**, 126, 127701.

[4] K.-H. Ahn, A. Hariki, K.-W. Lee, J. Kuneš, Phys. Rev. B **2019**, 99, 184432.

[5] S. Karube, T. Tanaka, D. Sugawara, N. Kadoguchi, M. Kohda, J. Nitta, Phys. Rev. Lett. **2022**, 129, 137201.

[6] A. Bose, N. J. Schreiber, R. Jain, D. -F. Shao, H. P. Nair, J. Sun, X. S. Zhang, D. A. Muller, E. Y. Tsymbal, D. G. Schlom, D. C. Ralph, Nat. Electron. **2022**, 5, 267-274.

[7] M. Sboui, H. Lachheb, M. Swaminathan, J.H. Pan, Cellulose **2022**, 29, 1189.

[8] A.V. Korotcov, Y.-S. Huang, K.-K. Tiong, D.-S. Tsai, J. Raman Spectrosc. **2007**, 38, 737.

[9] U. Balachandran, N.G. Eror, J. Solid State Chem. **1982**, 42, 276.

[10] P. Hohenberg, W. Kohn, Phys. Rev. **1964**, 136, B864-B871.

[11] G. Kresse, J. Hafner, Phys. Rev. B **1993**, 47, 558-561.

[12] G. Kresse, J. Furthmüller, Phys. Rev. B **1996**, 54, 11169.

[13] G. Kresse, D. Joubert, Phys. Rev. B **1999**, 59, 1758.

[14] J. P. Perdew, K. Burke, M. Ernzerhof, Phys. Rev. Lett. **1996**, 77, 3865-3868.

[15] V. I. Anisimov, J. Zaanen, O.K. Andersen, Phys. Rev. B **1991**, 44, 943.

[16] S. L. Dudarev, G. A. Botton, S. Y. Savrasov, C. Humphreys, A.P. Sutton, Phys. Rev. B **1998**, 57, 1505.
